# Two cyanobacterial species exhibit stress responses when grown together in visible light or far-red light

**DOI:** 10.1128/msphere.00251-24

**Published:** 2024-08-09

**Authors:** Ting-Shuo Nien, Ting-Hsuan Chan, Ying-Yang Li, Ting-So Liu, Yo-Jin Shiau, Ming-Yang Ho

**Affiliations:** 1 Department of Life Science, National Taiwan University, Taipei, Taiwan; 2 Institute of Plant Biology, National Taiwan University, Taipei, Taiwan; 3 Department of Bioenvironmental Systems Engineering, National Taiwan University, Taipei, Taiwan; University of Wisconsin-Madison, Madison, Wisconsin, USA

**Keywords:** cyanobacteria, coculture, far-red light photoacclimation (FaRLiP), negative interaction, transcriptomic analysis

## Abstract

Although most cyanobacteria grow in visible light (VL; *λ* = 400–700 nm), some cyanobacteria can also use far-red light (FRL; *λ* = 700–800 nm) for oxygenic photosynthesis by performing far-red light photoacclimation. These two types of cyanobacteria can be found in the same environment. However, how they respond to each other remains unknown. Here, we reveal that coculture stresses FRL-using *Chlorogloeopsis fritschii* PCC 9212 and VL-using *Synechocystis* sp. PCC 6803. No significant growth difference was found in *Synechocystis* sp. PCC 6803 between the coculture and the monoculture. Conversely, the growth of *Chlorogloeopsis fritschii* PCC 9212 was suppressed in VL under coculture. According to transcriptomic analysis, *Chlorogloeopsis fritschii* PCC 9212 in coculture shows low transcript levels of metabolic activities and high transcript levels of ion transporters, with the differences being more noticeable in VL than in FRL. The transcript levels of stress responses in coculture were likewise higher than in monoculture in *Synechocystis* sp. PCC 6803 under FRL. The low transcript level of metabolic activities in coculture or the inhibition of cyanobacterial growth indicates a possible negative interaction between these two cyanobacterial strains.

**IMPORTANCE:**

The interaction between two cyanobacterial species is the primary focus of this study. One species harvests visible light, while the other can harvest far-red and visible light. Prior research on cyanobacteria interaction concentrated on its interactions with algal, coral, and fungal species. Interactions between cyanobacterial species were, nevertheless, rarely discussed. Thus, we characterized the interaction between two cyanobacterial species, one capable of photosynthesis using far-red light and the other not. Through experimental and bioinformatic approaches, we demonstrate that when one cyanobacterium thrives under optimal light conditions, it stresses the remaining cyanobacterial species. We contribute to an ecological understanding of these two kinds of cyanobacteria distribution patterns. Cyanobacteria that utilize far-red light probably disperse in environments with limited visible light to avoid competition with other cyanobacteria. From a biotechnological standpoint, this study suggests that the simultaneous cultivation of two cyanobacterial species in large-scale cultivation facilities may reduce the overall biomass yield.

## INTRODUCTION

Cyanobacteria are unique photoautotrophic gram-negative bacteria that use light energy to perform photosynthesis to produce energy and reductant (NADPH) to fix carbon (1, 2). Most cyanobacteria, algae, and plants use wavelengths of light between 400 nm and 700 nm, known as visible light (VL), for photosynthesis (2, 3). However, VL is not always available due to factors such as shade from plants, absorption by other photoautotrophs, or inaccessibility. For instance, VL may be limited at the bottom of forest canopies, deep layers in microbial mats, or caves (4–7). To overcome this limitation, some cyanobacteria in such environments developed a mechanism called far-red light photoacclimation (FaRLiP), which allows them to utilize far-red light (FRL) with wavelengths ranging from 700 nm to 800 nm for photosynthesis (8).

FaRLiP is controlled by a FaRLiP gene cluster consisting of multiple genes, which includes three regulator genes: *rfpA*, *rfpB*, and *rfpC* (4, 8, 9). When the genes in the FaRLiP gene cluster are expressed, they enable cyanobacteria to remodel the structure of photosystem II (PSII), photosystem I (PSI), and the phycobilisome (PBS), and produce chlorophyll *d* and *f* to absorb FRL for photosynthesis (2, 4, 8, 10). Ultimately, this acclimation helps them survive and thrive in places where VL is scarce.

FRL-using cyanobacteria can be found in various regions around the world, including terrestrial environments such as subtropical rainforests, stromatolites, and caves, as well as aquatic environments such as freshwater lakes and coastal marine areas (2, 4, 7, 10–14). Some of these cyanobacteria are also present in microbial mats located in hot springs or on beach rocks, residing in deeper layers beneath other photoautotrophs on the surface that rely solely on VL for photosynthesis (6, 15). Due to the ability of FRL to penetrate deeper into the mats than VL, the proportion of FRL to VL increases with depth, allowing these cyanobacteria to utilize FRL for photosynthesis (6, 16).

Microorganisms often engage in interspecies interactions when growing in the same environment. These interactions can be positive or negative, depending on whether the growth of a particular species is promoted or suppressed by other coexisting species (17). For example, scramble competition (−,−) is a type of negative interaction that occurs when competitors in the same environment rapidly utilize limited resources to ensure their survival (17, 18). Conversely, amensalism (0,−) is another negative interaction in which one organism is adversely affected or inhibited while the other remains unaffected (17, 19). Moreover, allelopathy is a form of negative interaction in which one organism releases chemicals or compounds that inhibit the growth or development of another organism (20).

By metabolic interaction and the promotion effect of cocultured species, industrial-scale bioreactors can be optimized or produce bioproducts that are not feasible to produce using a single species (21). The coculture systems of cyanobacteria or microalgae with other microorganisms have been extensively utilized in hydrogen production and wastewater treatment (22, 23). The coculture of cyanobacteria that utilize FRL and cyanobacteria that utilize VL is likely to be of industrial value because these two types of cyanobacteria are capable of harvesting complementary spectra of sunlight from VL to FRL, potentially maximizing the total biomass yield. However, although these two types of cyanobacteria can be found in the same natural environment, it is still unclear whether they affect each other in the coculture conditions. In addition, despite our previous study demonstrating that the growth, pigment composition, and spectral properties of a FRL-using cyanobacterium are affected by a VL-using cyanobacterium in the coculture (16), it remains unknown how they respond in the gene expression level in the coculture. To address these questions, we selected a freshwater unicellular strain cyanobacterium, *Synechocystis* sp. PCC 6803 (Syn6803), as a representative of VL-using cyanobacteria in this study because it is one of the most studied cyanobacterial model strains (24). For FRL-using cyanobacterium, we chose a freshwater filamentous cyanobacterium, *Chlorogloeopsis fritschii* PCC 9212 (Cf9212), as a representative given that it is one of the most well-studied strains that can perform FaRLiP (4, 25–27). Although no specific research article explicitly states that strains from these two genera were isolated from the same environment, they probably coexist in comparable environments—for example, *Chlorogloeopsis fritschii* PCC 6912 was isolated from soil, whereas *Synechocystis* sp. PCC 7509 was obtained through rock scraping (28).

In this study, Syn6803 and Cf9212 were first grown as monoculture or coculture in liquid culture under VL or FRL. Second, the cells from monoculture and coculture were harvested, and Syn6803 and Cf9212 were separated. Finally, the collected cells were used for growth, pigment, and transcriptomic analyses. While growth and pigment analyses provide insight into how coculture was affected at the physiological level, the transcriptomic data reveal the gene expression affected by the coculture. Our research illustrates the correlation between cyanobacteria that utilize VL and those that utilize FRL, as well as how these two cyanobacterial strains interact.

## MATERIALS AND METHODS

### Cyanobacterial strains and growth conditions

**Fig 1:**
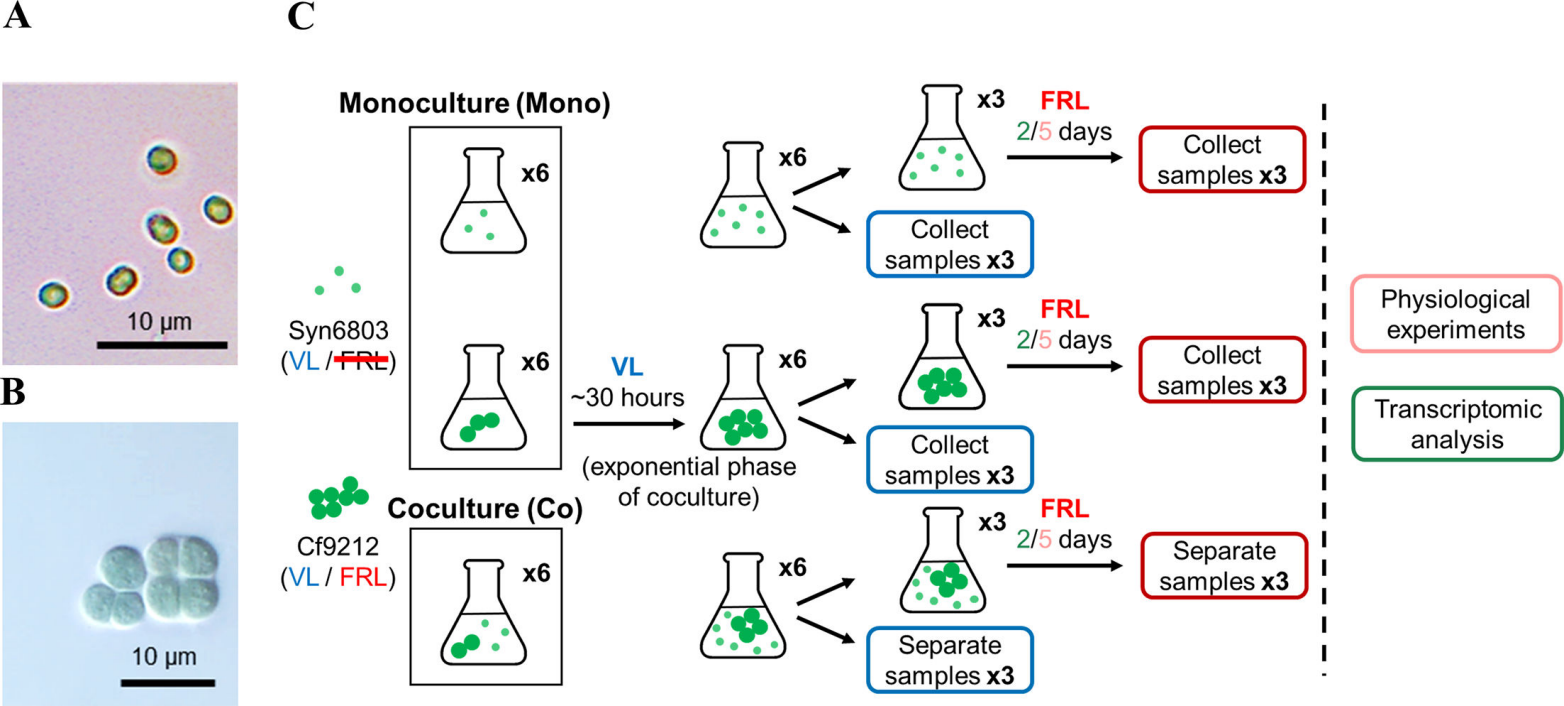
Microscopic images and experimental design. Microscopic images of (**A**) *Synechocystis* sp. PCC 6803 (Syn6803) and (**B**) *Chlorogloeopsis fritschii* PCC 9212 (Cf9212). Scale bars indicate 10 µm. (**C**) A schematic diagram illustrating the experimental design in this study. Light green dots represent Syn6803, which exclusively utilizes visible light for photosynthesis. Dark green solid circles represent Cf9212, which utilizes VL or FRL for photosynthesis. VL: visible light. FRL: far-red light. The methods employed in the study are shown to the right of the vertical dashed line.

The glucose-tolerant cyanobacterium Syn6803 was gifted from Dr. Hsiu-An Chu, Academia Sinica, Taiwan (Fig. 1A) (29). Cyanobacterium Cf9212 was obtained from the Pasteur Culture Collection (https://www.pasteur.fr/en/pcc) (Fig. 1B). Syn6803 and Cf9212 were grown in B-HEPES medium (30) in 100 mL flasks and stirred at 120 rpm in a 30°C incubator supplied with 1% (vol/vol) CO_2_. VL was provided by a combination of blue (400–490 nm), green (490–590 nm), and red (590–700 nm) LED light (167 µmol photons m^−2^ s^−1^ total irradiance); FRL was produced using LED light centered at 730 nm (90 µmol photons m^−2^ s^−1^) as described previously (16). In all experiments, Syn6803 and Cf9212 were either grown as monoculture or coculture. Prior to growth and pigment measurements, RNA extraction for transcriptomic analysis, and measurement of growth curves, Syn6803 and Cf9212 were cultured in B-HEPES medium under VL until the late exponential phase (OD_750_ = 0.6–0.8). Subsequently, the final concentration of both Syn6803 and Cf9212 was adjusted to OD_750_ = 0.025 for monoculture. Syn6803 and Cf9212, each with an OD_750_ of 0.025, were combined in coculture, resulting in a final OD_750_ of 0.05. About 0.45 mg of Syn6803 and 1.46 mg of Cf9212 were mixed for the coculture (Fig. S1). OD_750_ was measured with a Cary 60 UV-Vis spectrometer (Agilent, CA, USA). Cells grown under VL were collected after 30 hours for the growth and pigment measurements. Cells grown under FRL were initially grown in VL for 30 hours and then transferred to FRL for 5 days prior to collection (Fig. 1C). For transcriptomic analysis, cells grown under VL were collected after 30 hours. Similarly, cells grown under FRL were first grown in VL for 30 hours and then transferred to FRL for 2 days before collection (Fig. 1C).

### Syn6803 and Cf9212 cells’ collection from coculture and monoculture

Coculture samples were transferred to 50 mL falcon tubes to separate and collect Syn6803 and Cf9212. Because Cf9212 cells showed less aggregation when grown under FRL than under VL, Cf9212 were collected using low-speed centrifugation at 10 × *g* for samples from VL or 140 × *g* for samples from FRL by a himac high-speed refrigerated centrifuge (Hitachi, Tokyo, Japan) for 3 minutes at 4°C, followed by standing still on ice for 3 minutes. While Syn6803 remained suspended in the medium, Cf9212 precipitated to the bottom. After the supernatant containing Syn6803 was transferred to new falcon tubes, the remaining Cf9212 cells were washed with 20 mL of ddH_2_O at 4°C and precipitated again through low-speed centrifugation at 10 × *g* for samples from VL or 140 × *g* for samples from FRL for 3 minutes. The cells were washed four times to remove the remaining Syn6803 in the Cf9212 cell culture.

Meanwhile, the supernatant containing Syn6803 was centrifuged at 10 × *g* for samples from VL or 140 × *g* for samples from FRL for 3 minutes at 4°C, followed by standing still on ice for 3 minutes, and the supernatant was transferred to new falcon tubes. The centrifuge was repeated twice to remove the remaining Cf9212 in the Syn6803 cell culture. Finally, after separation, the cell cultures of Cf9212 and Syn6803 were pelleted by centrifugation at 9,190 × *g* at 4°C for 3 minutes for growth and pigment measurements or stored at −80°C for RNA extraction. For the samples from monoculture, the monoculture cells for growth and pigment measurements were collected using the same method as for the coculture, and the cells for RNA extraction were directly pelleted by centrifugation at 9,190 × *g* at 4°C for 3 minutes.

### pH value measurement, pigment extraction, and dry weight measurement

pH values of each culture were measured at the beginning of culturing, after 30 hours in VL, and after 5 days in FRL by a SARTORIUS PB-10 pH meter (Sartorius, Goettingen, Germany). For pigment extraction, 1 mL of Syn6803 or Cf9212 cells was centrifuged at 6,200 × *g* for 2 minutes with a Fresco 21 microcentrifuge (Thermo Fisher Scientific, MA, USA) and washed with 10 mM Tris-HCl pH = 8.0 buffer. The cells were resuspended with 30 µL of ddH_2_O and 970 µL of MeOH and then transferred to 2 mL screw tubes containing 100 µL of 0.1 mm glass beads (BioSpec Products, OK, USA). The screw tubes were agitated by a Model 607 Mini Beadbeater 16 machine for 10 seconds at 3,500 rpm (BioSpec Products, OK, USA) to extract pigments. The cell debris was removed by centrifugation at 16,200 × *g* for 3 minutes, and OD_750_, OD_664_, and OD_461 nm_ of the resulting supernatant were measured with a Cary 60 UV-Vis spectrometer (Agilent, CA, USA). The amount of chlorophyll and carotenoid was calculated from the following equations (31):


$$[chlorophyll\;a\;(\mu g\;mL^{-1})]=(OD_{664}-OD_{750})\times 11.92\;[carotenoids\;(\mu g\;mL^{-1})]=[(OD_{461}-OD_{750})-0.046\times (OD_{664}-OD_{750})]\times 4$$

For the measurement of dry weight, cells were centrifuged at 6,200 × *g* with a himac high-speed refrigerated centrifuge (Hitachi, Tokyo, Japan) for 2 minutes and dried in a DFO 80 oven (YIH DER, New Taipei City, Taiwan) set at 60°C for over 3 days (32).

### RNA extraction and transcriptional profiling with RNA sequencing

Total RNA from monoculture and coculture cells was extracted using the previously described method (25). Briefly, the cyanobacterial cells were resuspended with 10 mM Tris–HCl, pH 8.0, placed at room temperature for 2 minutes, and transferred into 2 mL screw tubes containing 200 µL of 0.1 mm glass beads (BioSpec Products, OK, USA) and 400 µL of acidic phenol:chloroform solution (1:1 [vol/vol], pH 4.3). Each sample was agitated by a Model 607 Mini Beadbeater 16 machine (BioSpec Products, OK, USA) for 10 seconds at 3,500 rpm and then placed on ice for 1 minute. This process was repeated three times. After the final agitation step, the samples were centrifuged at 10,000 × *g* at 22°C for 2 minutes to collect the aqueous phase of the samples. The aqueous phase was extracted once more with 300 µL of acidic phenol:chloroform solution (1:1 [vol/vol], pH 4.3) and extracted again with 300 µL of chloroform:isoamyl alcohol (24:1 [vol/vol]). The RNA was precipitated by adding 20 µL of 3.0 M sodium acetate, pH 5.2, and 500 µL of 4°C 100% ethanol and stored at −80°C for 1 hour. The precipitated RNA was centrifuged at 16,500 × *g* at 4°C for 30 minutes, washed with 4°C 75% ethanol twice, and then dried by a CVE-2000 evaporator (EYELA, Tokyo, Japan) for 3 minutes. RNA pellets were treated with DNase using the RNase-Free DNase Set (Qiagen, Hilden, Germany) according to the manufacturer’s instructions, with the addition of 3 µL of RNase inhibitor. The treatment time was extended to 2 hours to ensure complete digestion of the remaining DNA. Following the manufacturer’s instructions, the DNase-treated RNA was purified using the High Pure RNA Isolation Kit (Roche, Basel, Switzerland). The rRNA was removed, and the cDNA library was constructed by Ribo-Zero Plus rRNA Depletion Kit (Illumina, CA, USA) following the manufacturer’s instructions. Sequencing was performed using an Illumina NovaSeq6000 platform (Illumina, CA, USA) in pair-end 150 bp mode. Reverse transcription PCR (RT-PCR) was performed on total RNA using a ProtoScript II First Strand cDNA Synthesis Kit, following the manufacturer’s instructions (NEB, MA, USA). The primers were designed to amplify ~200 bp regions of selected genes, while 16S rRNA was chosen as a housekeeping gene for loading control. The primers used for RT-PCR are listed in Table S1.

### Analysis of transcriptomic data

The fastq files obtained from RNA sequencing were processed using fastp v0.23.2 with default parameters for adapter trimming and quality control, except for a minimum Phred quality score of 25 (33). In addition, base correction in the overlapping regions of the paired-end reads was enabled to ensure high-quality data. The remaining rRNA in the trimmed fastq files was removed by BBDuk with Kmer length set as 21 to find the rRNA (BBMap version 38.95, http://jgi.doe.gov/data-and-tools/bb-tools/). The BBduk-trimmed fastq files were mapped against the genome using STAR v2.7.9a with the default setting (34), and the resulting alignment files in bam format were sorted by Samtools index v1.9 (35). Differences in gene expression were analyzed using FeatureCounts v2.0.3 with default settings, except for specifying input data containing paired-end reads and assigning all reads to overlap meta-features (36). The resulting gene count data were then processed using DESeq2 v1.38.2 with default settings to identify differentially expressed genes (DEGs), wth log2|fold change| > 1 and adjusted *P*-value < 0.05 (37). Heatmaps were generated based on the read counts of the DEGs. The read counts were normalized as transcripts per million (TPM) to account for gene length and sequencing depth differences. The normalized read counts of each gene in Syn6803 and Cf9212 are listed in Tables S2 and S3.

The DEGs were then annotated by two methods. In the first method, Blast2Go v5.2.5 was applied by inputting the DNA sequence of all the genes in Syn6803 or Cf9212 (38). The input sequences were compared with the NCBI and InterPro databases and annotated with gene ontology (GO) terms in the GO database. Finally, the functional annotations that were statistically significant were shown by comparing the DEG lists with the total gene through enrichment analysis using Fisher’s exact test. The functional annotations were selected to show in each figure based on the least false discovery rate (FDR). In the second method, the DEG lists were inputted into the “multiple proteins searching” in STRING (https://string-db.org/) and compared with the STRING database (39). The RefSeq locus tags for the DEGs are converted to GenBank locus tags by CyanoIdMapping (http://www.cyanoomics.cn/lz/id-mapping) for the convenience of annotation searching (40). Unique annotations from STRING that were not identified by Blast2Go were reported.

### Measurement of nitrate and sulfate concentration

Syn6803 and Cf9212 were grown in a B-HEPES medium until the late exponential phase (OD_750_ = 0.6–0.8). Subsequently, they were diluted to OD_750_ = 0.025 for the monoculture, after which the two strains were mixed for the coculture. Each monoculture and coculture sample, carried out in three replicates, was then grown under VL for 30 hours and transferred to FRL for 5 days. Five milliliters of cell cultures from each sample was collected after being grown under VL for 30 hours and under FRL for 2 and 5 days. The supernatant was collected using the Himac high-speed refrigerated centrifuge (Hitachi, Tokyo, Japan) at 6,200 × *g* for 2 minutes and filtered through 0.22 µm filters. Using an Eco IC ion chromatography system (Metrohm AG, Herisau, Switzerland), the filtered supernatant was appropriately diluted to measure nitrate and sulfate concentrations. The measured values from each sample in microsiemens per centimeter (μS/cm) were converted to ppm using calibration curves made from standard samples containing 1, 10, and 100 ppm of nitrate and sulfate.

## RESULTS

### The growth of Cf9212 was suppressed when cocultured with Syn6803 under VL

The growth curves of Syn6803 monoculture, Cf9212 monoculture, and the coculture (Syn6803 + Cf9212) grown under VL and FRL were compared (Fig. S2). The doubling time of each cell culture was calculated based on the growth curves in the exponential growth phase. Under VL conditions, no significant difference in doubling time was observed between the Syn6803 monoculture (8.3 ± 0.6 h) (16) and the coculture (7.5 ± 0.2 h). However, the Cf9212 monoculture exhibited a doubling time five times longer (37.7 ± 0.5 h) than the Syn6803 monoculture and coculture under VL. By contrast, under FRL conditions, the doubling time of Cf9212 monoculture (63.6 ± 4.3 h) was shorter than that in the coculture (73.7 ± 2.7 h). Nevertheless, the Syn6803 monoculture displayed a doubling time five times longer (362.1 ± 64.0 h) (16) than the Cf9212 monoculture and the coculture under FRL.

**Fig 2:**
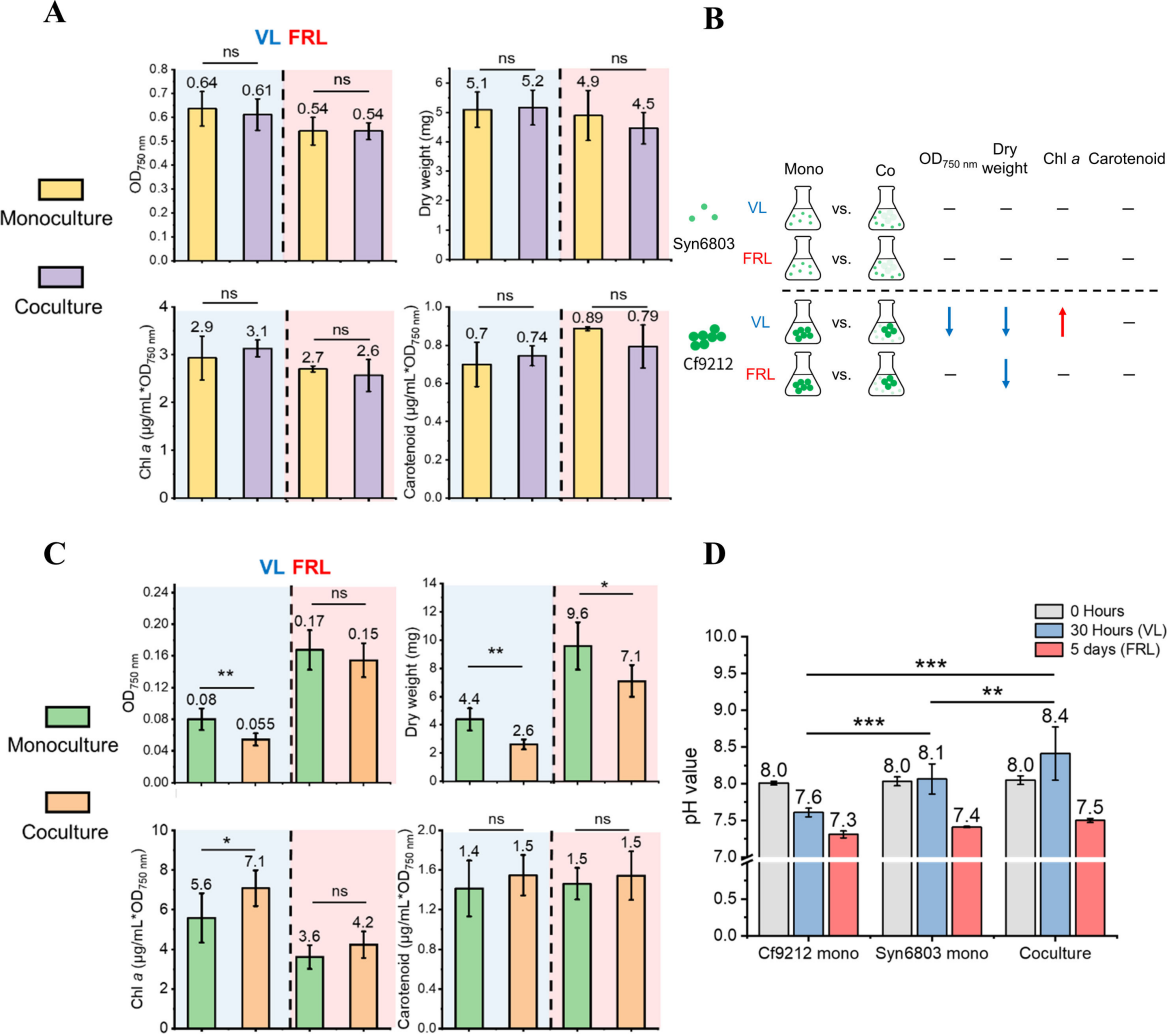
Growth and pigment measurements of (**A**) Syn6803 and (**C**) Cf9212 in monoculture (light yellow in Syn6803, green in Cf9212) and coculture (purple in Syn6803, orange in Cf9212) under VL (blue background) and FRL (red background). OD_750_, dry weight, chlorophyll *a* (Chl *a*) per OD_750_, and carotenoid per OD_750_ were measured. (**B**) A schematic diagram summarizing the results in panels A and C. The red arrow indicates the value is significantly higher in coculture than in monoculture, the blue arrows indicate the values are significantly lower in coculture than in monoculture, and the dashed lines mean there is no significant difference between monoculture and coculture. (**D**) pH values of Cf9212 monoculture, Syn6803 monoculture, and coculture were measured at the start of culturing (0 hours), after 30 hours in VL, and after 5 days in FRL. Mann-Whitney *U* test was conducted based on at least three replicates. Statistical significance levels are indicated as follows: *P < 0.05, **P < 0.01, ***P < 0.001; ns, non-significant. Mono, monoculture, Co, coculture.

To further investigate the coculture effect on the growth of cyanobacteria in VL and FRL, we cultured Syn6803 and Cf9212 as monocultures or cocultures for growth and pigment measurements. The cultures were maintained under VL conditions for 30 hours until the coculture reached the late exponential phase. Alternatively, after 30 hours of growth under VL conditions, the cultures were transferred to FRL conditions for 5 days. The results show that when Syn6803 was grown under VL, there were no significant differences in the four measurements (OD_750_, dry weight, Chl *a* per OD_750_, and carotenoid per OD_750_) between monoculture and coculture. Similarly, under FRL conditions, no significant differences were observed between monoculture and coculture (Fig. 2A and B). On the contrary, the measured growth characteristics of Cf9212 under VL exhibited significant variations between monoculture and coculture. In particular, the Chl *a* per OD_750_ was significantly higher in coculture than in monoculture, whereas the OD_750_ and dry weight were significantly lower in coculture compared to monoculture. Under FRL conditions, only dry weight was significantly lower in coculture than in monoculture (Fig. 2B and C).

In addition, pH measurements were taken at the start of the experiment, 30 hours into the growth of the cultures under VL, and 5 days into the experiment under FRL. The findings indicate that when subjected to VL conditions, the pH levels of the Syn6803 monoculture and coculture increased to 8.4 and 8.1, respectively. By contrast, the pH levels of the Cf9212 monoculture dropped from 8.0 to 7.6. In addition, under VL, the pH of the coculture was significantly higher than that of the Syn6803 and Cf9212 monocultures. However, the pH values decreased in all three conditions once the cultures were transferred to the FRL condition (Fig. 2D).

Overall, the growth and pigment measurements demonstrated that coculture minimally affected Syn6803 growth under both VL and FRL conditions. However, coculture significantly affected the growth of Cf9212 under VL conditions (Fig. 2B). The differences in pH values between coculture and monoculture under VL further highlight the distinctions between the two conditions.

### Transcriptomic data from Syn6803 grown as monoculture or coculture under VL or FRL were clustered into four groups

To understand the coculture effect on the growth of cyanobacteria in VL and FRL at the transcript level, we performed transcriptomic analysis on monoculture and coculture cells of Syn6803 and Cf9212. Before RNA extraction and sequencing, the monoculture and coculture were grown under VL for 30 hours (VL group) or under VL for 30 hours, followed by transferring the cultures to FRL conditions for 2 days (FRL group), as 2 days in FRL could induce FaRLiP strongly in Cf9212 (25). There were 12 RNAseq data sets derived from Syn6803 in four different conditions, including monoculture under VL (Mo_VL), coculture under VL (Co_VL), monoculture under FRL (Mo_FRL), and coculture under FRL (Co_FRL), each with three replicates. On average, 57.6 million sequence reads were obtained from each Syn6803 RNAseq raw data set. After removing low-quality reads, rRNA sequences, and adapters, an average of 43.7 million sequence reads remained and were mapped to the reference genome. Of these, an average of 40.5 million sequence reads, which accounted for 93.0% of the total reads, were uniquely mapped. Finally, an average of 38.6 million sequence reads were successfully assigned to gene annotations.

**Fig 3:**
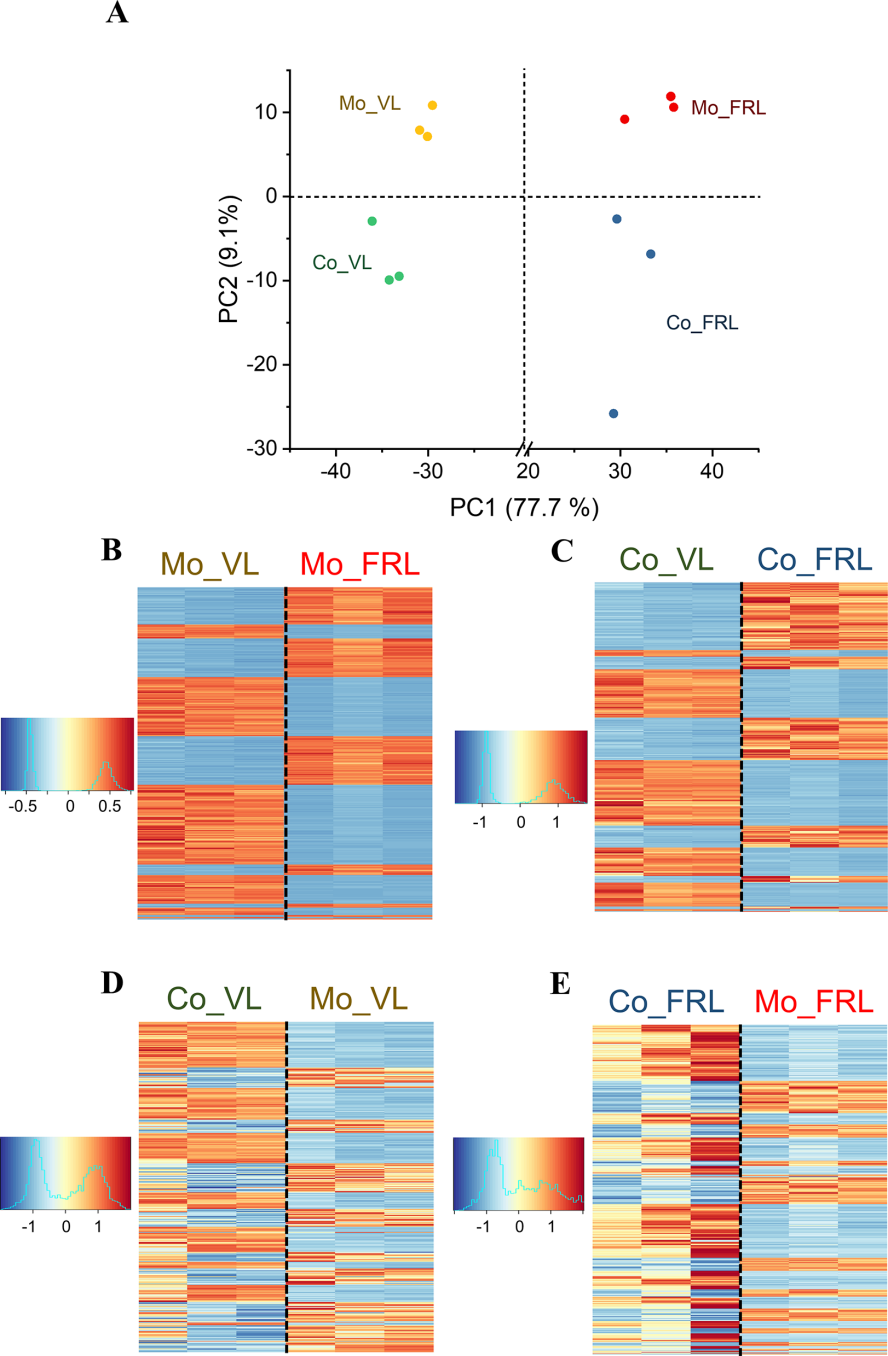
Transcriptomic analyses of Syn6803 under monoculture or coculture condition in VL or FRL. (**A**) PCA was performed on the transcriptomic profiles of Syn6803. Each dot represents RNA-seq data obtained from Syn6803 monoculture (Mo) or coculture (Co) under VL or FRL. The experiment was conducted in triplicate for each condition. Heatmaps of the top 500 DEGs are shown for (**B**) Syn6803 monoculture and (**C**) Syn6803 coculture under VL or FRL conditions. (**D**) Comparison of coculture and monoculture of Syn6803 in VL and (**E**) comparison of coculture and monoculture of Syn6803 in FRL. DEGs were selected based on the smallest adjusted *P*-values from the RNA-seq data comparison. The color scale represents the z-score values of gene expression levels, with red indicating upregulation and blue indicating downregulation in the comparison. Each row corresponds to an individual gene, and the columns represent three biological replicates for each condition.

Principal component analysis (PCA) was applied to assess the similarity of each sample (Fig. 3A). The results show that principle component 1 (PC1) explained 77.7% of the variance, which separated FRL samples from VL samples with a clear distance. Principle component 2 (PC2) explained 9.1% of the variance and distinguished coculture samples from monoculture samples. To further examine the disparity of gene expression across different conditions, we generated heatmaps using the top 500 DEGs in Syn6803, selected based on the smallest adjusted *P*-values. The heatmaps reveal distinct expression patterns between the FRL and VL samples in monoculture and coculture (Fig. 3B and C). In addition, significant differences in gene expression patterns were observed between Syn6803 monoculture and coculture samples under both VL and FRL (Fig. 3D and E).

### FRL affected the transcript level of the genes related to metabolic processes in Syn6803 monoculture

To investigate the effect of FRL on Syn6803 growth, we analyzed the gene expression profiles of Syn6803 monoculture grown under FRL and VL conditions. The volcano plot shows that in FRL, 569 genes were upregulated (red dots), and 606 genes were downregulated (blue dots), with log2|fold change| > 1 and adjusted *P*-value < 0.05 (Fig. S3A). Among the genes that were the highest upregulated, some were related to phycobilisome degradation (*nblA*) (log2 fold change increased by 3.2) and the type II secretion system (log2 fold change increased by 3.4). On the other hand, the top five downregulated genes were mainly related to ribosomal protein (log2 fold change decreased by 4.4) (Fig. S3B).

To gain insight into the functional roles of the genes differentially expressed in Syn6803 under FRL, we performed functional enrichment analysis on the DEGs through Blast2GO and STRING. The upregulated genes in FRL revealed few functional annotations. Specifically, these genes were found to be involved only in the sigma factor activity in the molecular function category. They were associated with a STRING cluster related to o-antigen nucleotide sugar biosynthesis (Fig. S3C). Conversely, downregulated genes exhibited a greater quantity of functional annotations in FRL compared to the upregulated genes. In the biological process category, these genes were related to diverse metabolic processes, such as amide, peptide, carboxylic acid biosynthesis, and photosynthesis. The molecular function category included translation regulator activity, and the cellular component category included ribosome and thylakoid. In addition, these genes were significantly enriched in a STRING cluster related to the phycobilisome (Fig. S3D). The heatmap also reveals that the transcript levels of genes related to amide biosynthesis were significantly lower in FRL than in VL (Fig. S3E; Table S4). Furthermore, one of the genes related to amide biosynthesis, *rpl16* (SGL_RS05815), which encodes the 50S ribosomal protein L16, was selected for RT-PCR. The results confirm that the relative expression level of *rpl16* was significantly lower in FRL than in VL (Fig. S4A and B). Overall, the results indicate that the transcript levels related to metabolic processes decrease in Syn6803 when transferred to FRL.

### Coculture affected the transcript level of the genes related to protein folding and secretion in Syn6803 grown under FRL

To investigate the transcriptional change of coculture to Syn6803, we compared the gene expression of Syn6803 grown in coculture and monoculture. Under VL condition, the volcano plot shows that 50 genes were upregulated (red dots), and 12 genes were downregulated (blue dots) in coculture, with log2|fold change| > 1 and adjusted *P*-value < 0.05 (Fig. S5A). The top-upregulated genes were those related to ion-translocating P-type ATPase (log2 fold change increased by 2.1) and high light-inducible protein (log2 fold change increased by 2.0). On the contrary, some top-downregulated genes encode phycocyanin subunits (log2 fold change decreased by 1.2) (Fig. S5B). Functional enrichment analysis of the DEGs in Syn6803 grown under VL revealed some downregulated genes were related to the phycobilisome. Some upregulated genes were also associated with zinc transport under coculture conditions (Fig. S5C).

**Fig 4:**
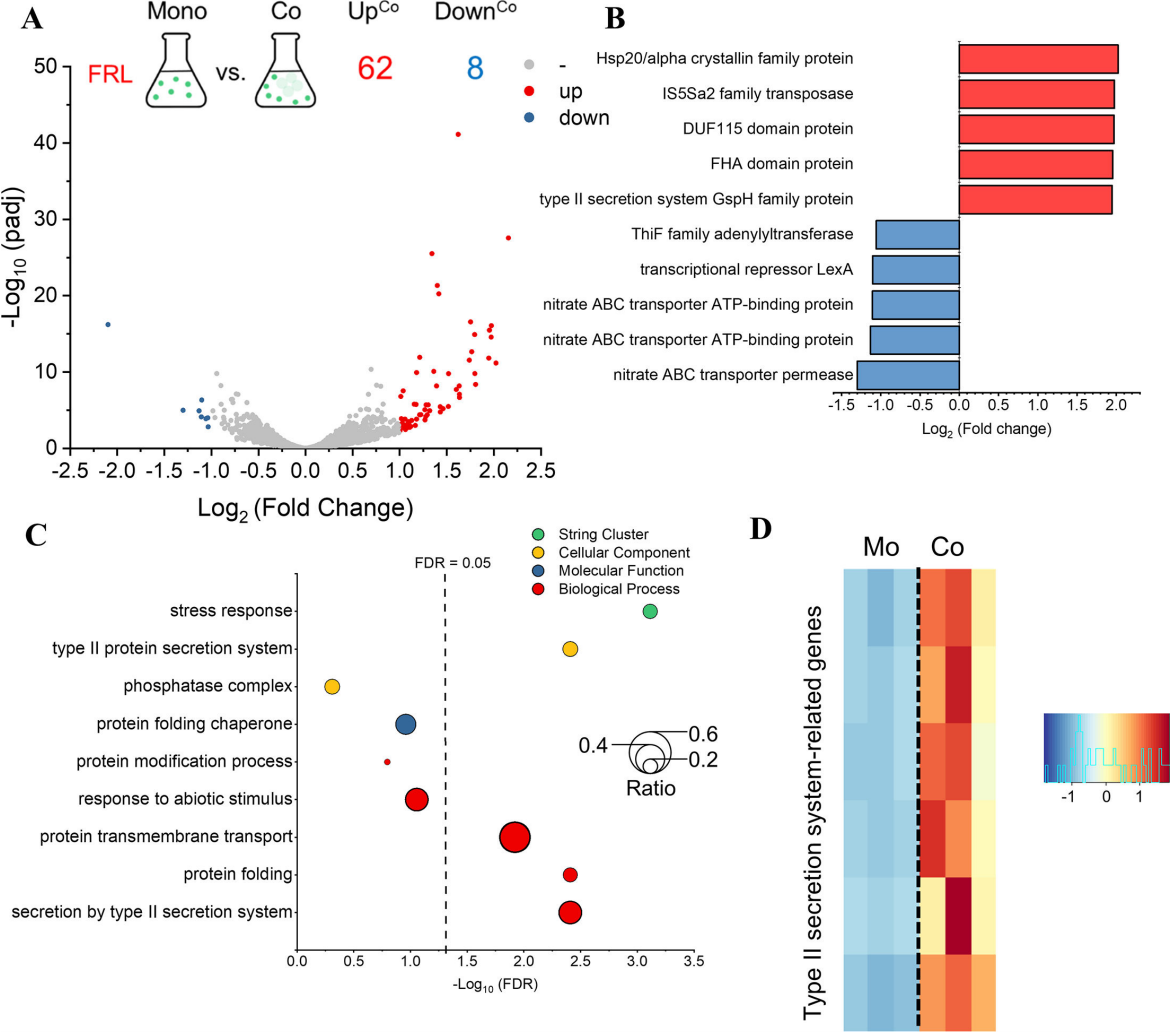
Transcriptomic profiles and gene ontology enrichment analysis of DEGs from the comparison between monoculture and coculture of Syn6803 in FRL. (**A**) Volcano plot of Syn6803 transcriptomic profiles in monoculture and coculture under FRL. Genes with adjusted *P*-values <0.05 and log2(fold change) >1 are highlighted in red (upregulated) and blue (downregulated), respectively. (**B**) Top 10 regulated DEGs from the comparison between monoculture and coculture of Syn6803 in FRL. Genes upregulated in coculture are marked in red, and genes downregulated in coculture are marked in blue. (**C**) Bubble plot of function annotations from upregulated genes in coculture. Each dot represents a function annotation from the GO database (red for biological process, blue for molecular function, and yellow for cellular component) or STRING database (green). The size of the dots is defined as the ratio of DEGs to total genes in each function annotation. The vertical dashed line represents a false discovery rate of 0.05. (**D**) Heatmap of type II protein secretion system-related genes. Only the differentially expressed genes are shown in the heatmap. The color scale represents the z-score values of the gene expression levels, with red indicating upregulation and blue indicating downregulation in coculture compared to monoculture. Mo, monoculture. Co, coculture.

Under FRL conditions, 62 genes were upregulated (red dots), and 8 genes were downregulated (blue dots) in Syn6803 coculture, with log2|fold change| > 1 and adjusted *P*-value < 0.05 (Fig. 4A). Some top-upregulated genes were related to heat shock protein (*hsp20*, log2 fold change increased by 2.0) and the type II protein secretion system (*gspH*, log2 fold change increased by 1.95). By contrast, some of the top downregulated genes were related to transcriptional repressor (*lexA*, log2 fold change decreased by 1.0) and nitrate ABC transporter (log2 fold change decreased by 1.3) (Fig. 4B).

Functional enrichment analysis of Syn6803 DEGs grown in FRL showed that upregulated genes in coculture are related to protein folding and protein transmembrane transport in the biological process category. Type II protein secretion system in the cellular component category and the stress response in the STRING cluster were also enriched in functional annotations in coculture (Fig. 4C). A heatmap of the genes related to the type II protein secretion system suggests that six genes were more highly expressed in coculture than monoculture under FRL (Fig. 4D; Table S5). Three genes, namely, *hsp20* (SGL_RS04040), *gspH* (SGL_RS07940), and *lexA* (SGL_RS08040), were selected for RT-PCR due to their high differential expression levels and their relevance to the functional annotations of protein folding, type II protein secretion system, and stress response (Fig. 4B and C). The results of RT-PCR were consistent with the transcriptomic analysis, showing that the relative expression levels of *hsp20* and *gspH* were significantly higher, and the relative expression level of *lexA* was significantly lower in coculture than in monoculture (Fig. S4). On the other hand, only nitrate transport in the biological process category was enriched in the downregulated genes (Fig. 4B).

In summary, more functional annotations were found in the DEGs of Syn6803 cocultured under FRL compared to VL, and coculture increased transcript levels of genes related to protein folding and secretion under FRL.

### Transcriptomic data from Cf9212 grown as monoculture or coculture under VL or FRL are clustered into four groups

There were 12 RNAseq data derived from Cf9212 under four culture conditions, including Mo_VL, Co_VL, Mo_FRL, and Co_FRL, each with three replicates. On average, 59.0 million sequence reads were obtained from each Cf9212 RNAseq raw data set. 52.8 million sequence reads remained on average after adapter trimming, rRNA sequence removal, and low-quality read removal were completed. These sequence reads were then mapped to the reference genome. An average of 46.8 million sequence reads were uniquely mapped, accounting for 86.8% of the total reads. Subsequently, on average, gene annotations were effectively assigned to 43.0 million sequence reads.

**Fig 5:**
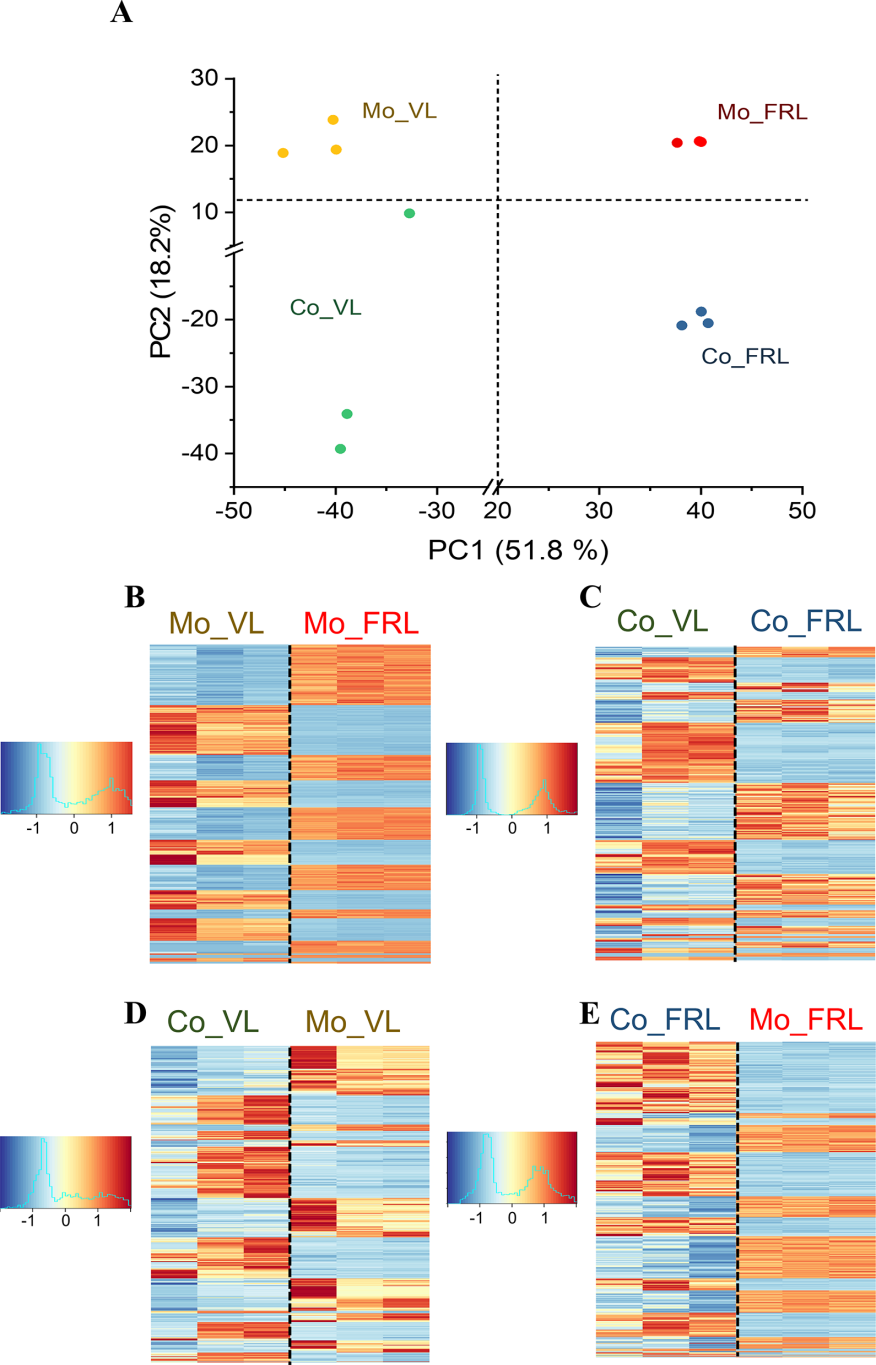
Transcriptomic analyses of Cf9212 under monoculture or coculture condition in VL or FRL. (**A**) PCA was performed on the transcriptomic profiles of Cf9212. Each dot represents RNA-seq data obtained from Cf9212 monoculture (Mo) or coculture (Co) under VL or FRL. The experiment was conducted in triplicate for each condition. Heatmaps of the top 500 DEGs are shown for (**B**) Cf9212 monoculture and (**C**) Cf9212 coculture under VL or FRL conditions. (**D**) Comparison of coculture and monoculture of Cf9212 in VL and (**E**) comparison of coculture and monoculture of Cf9212 in FRL. DEGs were selected based on the smallest adjusted *P*-values from the RNA-seq data comparison. The color scale represents the z-score values of gene expression levels, with red indicating upregulation and blue indicating downregulation in the two conditions. Each row corresponds to an individual gene, and the columns represent three biological replicates for each condition.

PCA was applied to assess the similarity of each sample (Fig. 5A). The results show that PC1 explained 51.8% of the variance, distinguishing FRL samples from VL samples. PC2 explained 18.2% of the variance, showing the separation between coculture and monoculture samples, although the replicates within the VL_Co condition were not as closely grouped as in other conditions. Heatmaps were generated to visualize the expression patterns of genes under different conditions. The results indicate distinct expression patterns of genes in FRL samples compared to VL, both in monoculture and coculture conditions (Fig. 5B and C). Moreover, under both VL and FRL conditions, gene expression patterns in coculture samples were distinct from those in monoculture (Fig. 5D and E). In summary, the results suggest that the RNA-seq data of Cf9212 can be clustered into four groups based on the experimental design.

### FRL triggered the expression of the FaRLiP gene cluster in Cf9212 monoculture

To investigate the impact of FRL on Cf9212 growth, we compared the gene expression profiles of Cf9212 monoculture grown under FRL and VL conditions. The volcano plot reveals that in FRL, 398 genes were upregulated (red dots), and 586 genes were downregulated (blue dots), with log2|fold change| > 1 and adjusted *P*-value < 0.05 (Fig. S6A). The top five upregulated genes were all in the FaRLiP gene cluster (log2 fold change increased by up to 15.5). On the other hand, some of the top-downregulated genes were related to ABC transporter (log2 fold change decreased by 4.6) and N-acetyltransferase (log2 fold change decreased by 4.4).

Functional enrichment analysis indicated that the upregulated genes were enriched in functional annotations related to photosynthesis. Specifically, the enriched annotations included photosynthesis (from the FaRLiP gene cluster) and electron transport chain in the biological process category, chlorophyll-binding in the molecular function category, thylakoid in the cellular component category, and nitrate transport in the STRING cluster (Fig. S6B). By contrast, the downregulated genes were enriched in functional annotations related to various metabolic processes, including amide, peptide, fatty acid biosynthesis, and photosynthesis (non-FaRLiP genes) in the biological process category. The downregulated genes were also enriched in the ribosome and o-antigen nucleotide sugar biosynthesis categories (Fig. S6C). The heatmap of the FaRLiP gene cluster further supports the finding that the genes in the FaRLiP gene cluster were significantly more expressed in FRL than in VL (Fig. S6D; Table S6). *psaA1* (UYE_RS0111400) and *psaA2* (UYE_RS0105795), two representative genes, were chosen for confirmation by RT-PCR analysis. Under VL, the expression level of *psaA1*, encoding a subunit of VL-type photosystem I, was higher than in FRL. By contrast, the expression level of *psaA2*, one of the genes in the FaRLiP gene cluster encoding a subunit of FRL-type photosystem I, was higher in FRL than in VL (Fig. S7). The results generally suggest that FRL activates genes comprising the FaRLiP gene cluster, whereas it inhibits genes linked to metabolic processes in Cf9212, which is consistent with previous findings (25).

### Coculture affected the transcript level of the genes related to ion transport and metabolic processes in Cf9212 in VL

**Fig 6:**
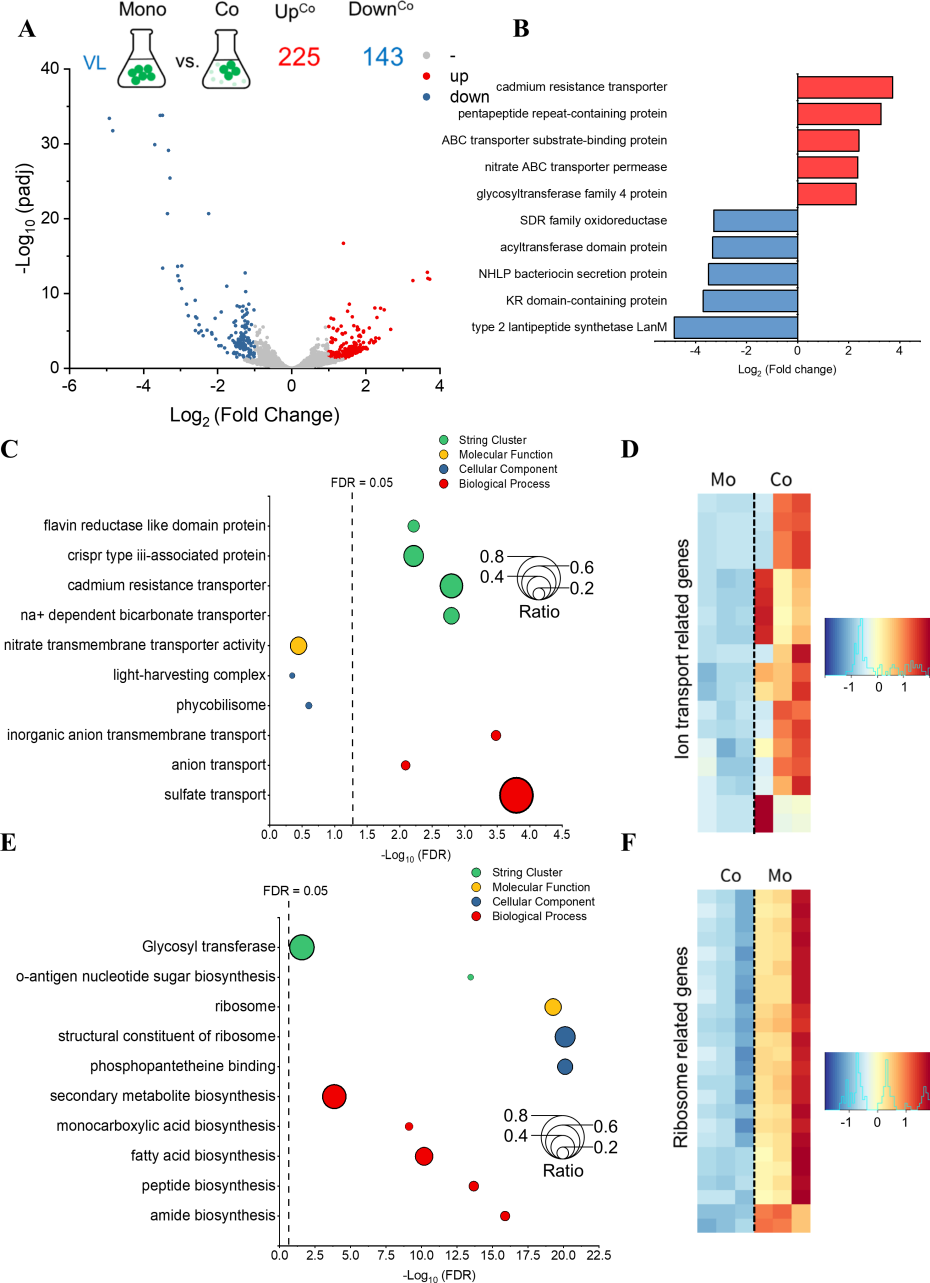
Transcriptomic profiles and gene ontology enrichment analysis of DEGs from the comparison between monoculture and coculture of Cf9212 in VL. (**A**) Volcano plot of Cf9212 transcriptomic profiles in monoculture and coculture under VL. Genes with adjusted *P*-values < 0.05 and log2(fold change) >1 are highlighted in red (upregulated) and blue (downregulated), respectively. (**B**) Top 10 regulated DEGs from the comparison between monoculture and coculture of Cf9212 in VL. Genes upregulated in coculture are marked in red, and genes downregulated in coculture are marked in blue. Bubble plot of function annotations from (**C**) upregulated genes and (**E**) downregulated genes in coculture. Each dot in panels C and E represents a function annotation from the GO database (red for biological process, blue for molecular function, and yellow for cellular component) or STRING database (green). The size of the dots is defined as the ratio of DEGs to total genes in each function annotation. The vertical dashed line represents a false discovery rate of 0.05. Heatmap of (**D**) ion transport-related genes from upregulated DEGs and (**F**) ribosome-related genes from downregulated DEGs. The z-score values of the gene expression levels are depicted on the color scale; in coculture, downregulation is indicated by blue, while upregulation is denoted by red, compared to monoculture. Mo, monoculture. Co, coculture.

To investigate the effect of coculture on the growth of Cf9212, we compared the gene expression of Cf9212 grown in coculture and monoculture. Under the VL condition, there were 225 upregulated genes and 143 downregulated genes in coculture, with log2|fold change| > 1 and adjusted *P*-value < 0.05 (Fig. 6A). Some top-upregulated genes were related to transporters, including cadmium resistance transporter (log2 fold change increased by 3.7) and nitrate ABC transporter (log2 fold change increased by 2.4). By contrast, some top-downregulated genes, such as acyltransferase domain protein and lantipeptide synthase (log2 fold change decreased by 3.5), might be involved in metabolic processes (Fig. 6B).

**Fig 7:**
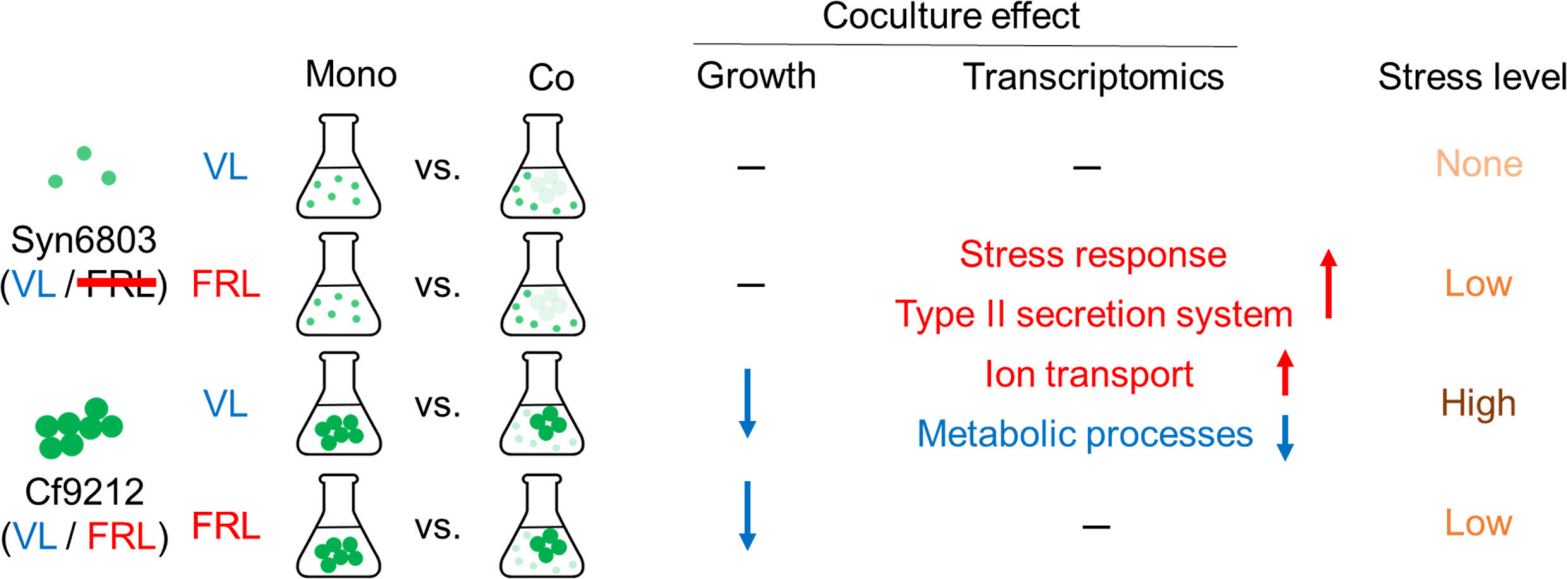
The schematic diagram illustrates the conclusions of this study.

Functional enrichment analysis suggested that the upregulated genes in the coculture under VL were enriched in functional annotations related to diverse transporter functions, including sulfate transporters in the biological process category, Na^+^-dependent bicarbonate transporters, and cadmium resistance transporters in STRING clusters (Fig. 6C). In addition, three genes encoding nitrate transporter subunits (UYE_RS0104885, UYE_RS0104890, and UYE_RS0104895) exhibited a higher expression level in coculture than in monoculture (Table S7). Moreover, CRISPR type-III-associated protein in a STRING cluster was also enriched among the upregulated genes (Fig. 6C). The heatmap of genes related to ion transporters, including sulfate transporters, bicarbonate transporters, and nitrate transporters, further demonstrated that the expression levels of these genes were significantly higher in coculture than in monoculture (Fig. 6C and D; Table S7). By contrast, the downregulated genes were enriched in functional annotations related to various metabolic processes, including amide, peptide, and fatty acid biosynthesis. Furthermore, these genes were enriched in the ribosome in the cellular component category, as well as glycosyl transferase and o-antigen nucleotide sugar biosynthesis in STRING clusters (Fig. 6E). The heatmap of ribosome-related genes also indicates that their expression levels were significantly lower in coculture compared to monoculture (Fig. 6F; Table S8). Furthermore, genes encoding the nitrate transporter (UYE_RS0104890) and *rpsQ* encoding the 30S ribosomal protein S17 (UYE_RS0128510) were selected for RT-PCR. The results confirm that while the relative expression level of the gene related to the nitrate transporter was significantly higher in coculture than in monoculture, the relative expression level of *rpsQ* was significantly lower in coculture than in monoculture (Fig. S7).

Transcriptomic data suggest that the transcript levels of ion transporters in Cf9212, including genes related to nitrate and sulfate transporters, were higher in coculture than in monoculture under VL (Fig. 6C; Table S7). To investigate whether the differential expression of ion transporters in coculture was caused by nutrient starvation, we measured nitrate and sulfate concentrations in the monoculture and coculture media after the cultures had grown under VL for 30 hours and were then transferred to FRL for 2 days and 5 days. The results show that the nitrate concentration in coculture decreased from 1025.9 ppm at 0 hours to 927.9 ppm after 30 hours under VL and further decreased to 866.2 ppm after being transferred to FRL for 5 days (Fig. S8A). Meanwhile, the sulfate concentration in coculture decreased from 22.3 ppm at 0 hours to 18.1 ppm after 30 hours under VL and then dropped to 17.3 ppm after being transferred to FRL for 5 days (Fig. S8B). The data suggest that the varied expression of ion transporters was not due to nutrient deprivation in coculture.

Under the FRL condition, there were 214 upregulated genes and 43 downregulated genes in coculture, with log2|fold change| > 1 and adjusted *P*-value < 0.05 (Fig. S9A). Some top-upregulated genes seemed to be associated with RNA binding and splicing, such as the TROVE domain-containing protein (log2 fold change increased by 4.5) and RtcB family protein (log2 fold change increased by 3.5). By contrast, some top-downregulated genes were associated with ABC transporter (log2 fold change decreased by 2.5) (Fig. S9B).

Functional enrichment analysis of the DEGs in the coculture under FRL revealed fewer functional annotations than in the coculture under VL. However, the upregulated genes were enriched with functional annotations related to microbial metabolism and the cadmium resistance transporter in the STRING clusters. The GO term “ion transmembrane transport” was also present in the biological process category, although not significantly enriched (Fig. S9C). Conversely, the downregulated genes were enriched with functional annotations related to ABC transporters, choline metabolic process, and fatty acid biosynthesis in the STRING clusters (Fig. S9D).

Overall, more functional annotations were identified in the DEGs of Cf9212 cocultured in VL than in FRL. Under VL, the transcript level of ion transporter-related genes was higher, while the expression of genes related to metabolic processes was lower in coculture than in monoculture.

## DISCUSSION

Our study is the first to describe how coculture could affect cyanobacteria acclimated to VL and FRL conditions using a transcriptomic approach. The VL-using cyanobacterium Syn6803 experienced intense stress in FRL and FRL in coculture, and the FRL-using cyanobacterium Cf9212 underwent strong stress in VL in coculture. In previous studies, Syn6803 has been grown in FRL to investigate the function of phytochromes, light-signal-driven RNA helicase, or proteorhodopsin (41–43). However, to our knowledge, only two studies have examined the effect of FRL on Syn6803 at the transcript level (44, 45). Since the growth of Syn6803 was strongly inhibited in FRL, FRL is a stress condition for Syn6803.

The transcript levels of sigma factor-related genes were notably elevated in our transcriptomic data for Syn6803 under FRL compared to VL. Conversely, the expression levels of genes linked to metabolic processes, such as peptide biosynthesis and photosynthesis, were substantially reduced in FRL compared to VL (Fig. S3C and D). These findings are similar to the prior study that investigated the response of Syn6803 to a transition from red light to FRL, which reported enhanced expression of genes related to sigma factors and reduced expression of genes involved in amino acid biosynthesis and photosynthesis (44). Moreover, the expression levels of several genes related to the nitrate transport system were significantly lower in FRL. These genes included *nrtA* (SGL_RS06595, log2 fold change = −2.9), *nrtB* (SGL_RS06590, log2 fold change = −3.2), *nrtC* (SGL_RS06585, log2 fold change = −2.8), and *nrtD* (SGL_RS06580, log2 fold change = −2.9) (Table S2). The expression levels of the *nrtABCD* operon were also reported to be decreased under FRL in another previous study that compared the transcriptomic profiles of Syn6803 under solar light and FRL (45). Sigma factors play a crucial role in regulating gene expression, enabling the initiation of transcription in bacteria (46). The expression of sigma factors is influenced by various environmental stimuli, including stress (47). In addition, the reduced expression level of metabolic processes under FRL may hinder the growth of Syn6803, further supporting the notion that FRL imposes stress on Syn6803 growth.

In addition to FRL, coculture may impose additional stress on Syn6803 under FRL conditions. The transcriptomic profiles of Syn6803 revealed significantly higher transcript levels of protein folding in coculture than in monoculture under FRL (Fig. 4C; Table S9). Furthermore, the transcript level of *sodB* (SGL_RS09370, log2 fold change = 1.63) was also significantly higher in coculture. Among the genes associated with the GO term “protein folding” are molecular chaperones such as *clpB* (SGL_RS11615, log2 fold change = 1.32), *groEL* (SGL_RS13680, log2 fold change = 1.23), *groES* (SGL_RS06165, log2 fold change = 1.18), *htpG* (SGL_RS18085, log2 fold change = 1.17), and *dnaK* (SGL_RS12750, log2 fold change = 1.10). Previous studies show that these chaperones are expressed under stress conditions, including heat stress and high light intensity, in Syn6803 (48, 49). *sodB* is a gene encoding superoxide dismutase, which can eliminate reactive oxygen species (ROS) (49). The higher expression of chaperones and ROS-related genes indicates that coculture becomes an additional stress factor under FRL. Nevertheless, when one considers the substantial stress that FRL already places on the development of Syn6803, the stress induced by coculture is comparatively less severe than that of FRL.

In the coculture of Cf9212 under VL, the transcript levels of transporters, including sulfate, nitrate, and bicarbonate transporters, were significantly higher than in monoculture (Fig. 6C). Moreover, nitrate and sulfate concentrations were significantly lower in the coculture than in the Cf9212 monoculture under VL and FRL conditions. Sulfate is a vital anion nutrient in cyanobacteria, responsible for protein and coenzyme synthesis, and plays a role in the photosynthetic membrane and photosystems (50). Nitrate is a nitrogen source in cyanobacteria for protein and nucleic acid synthesis. Moreover, bicarbonate is an essential carbon source for photosynthesis and growth (51, 52). Prior research has demonstrated that carbon, sulfate, and nitrogen limitations can increase the expression of sulfate, nitrate, and bicarbonate transporters in cyanobacteria (53–55). Some studies also indicated that cyanobacteria growth is inhibited under sulfate and nitrate concentrations below 2.9 ppm and 1.2 ppm, respectively (56, 57). However, in our study, the nutrient limitation was unlikely because both nitrate (i.e., 866–1,026 ppm) and sulfate (i.e., 17.3–22.3 ppm) in the coculture medium were sufficient when the time cells were harvested (Fig. S8). Moreover, the growth of Cf9212 and Syn6803 monocultures can ultimately reach an OD_750_ above 3.0 (16, 26), which is higher than the value (OD_750_ ~0.8) when the coculture cells were harvested (Fig. 2A and B). Since nutrients are sufficient, the increased transcript levels of transporters in coculture may not be attributed to nutrient starvation. This observation may be elucidated through a view of scramble competition, a phenomenon in which competitors utilize limited resources rapidly to ensure their survival (18). For instance, some bacteria secrete high-affinity siderophores to acquire extracellular iron, which affects the iron uptake of other bacteria secreting low-affinity siderophores (18, 58). Scramble competition has also been observed in the root competition among plants, which results in the depletion of soil resources due to the competition for nutrients (59). Cf9212 might also experience scramble competition in the coculture, which shows increased transcript levels of transporters to obtain additional nutrients and competitive advantages.

Furthermore, the transcript levels of metabolic processes and the genes encoding ribosomal protein were significantly lower in the Cf9212 coculture than in the monoculture in VL (Fig. 6D). A previous study has demonstrated a similar phenomenon in a bloom-forming cyanobacterium, *Microcystis aeruginosa* when cocultured with an algicidal bacterium, *Brevibacillus laterosporus*. The investigation mentioned above shows the suppression of *M. aeruginosa* growth and downregulation of multiple genes associated with amino acid and carbohydrate metabolism, suggesting the initiation of a stress response (60). It is possible that Cf9212 also underwent growth stress when cocultured with Syn6803. Syn6803 potentially induces stress on Cf9212 via an unidentified mechanism, which leads to the suppression of ribosomal protein genes and metabolic processes. Consequently, the dry weight and growth rate of Cf9212 were adversely affected, and its cellular content and OD_750_ decreased.

While numerous studies have examined the interspecies interaction between cyanobacteria and other microorganisms (61–64), our report represents the first to investigate an interaction between cyanobacteria that utilize FRL and those that utilize VL, suggesting a possible negative interaction between the VL-using cyanobacterium Syn6803 and the FRL-using cyanobacteria Cf9212 under VL conditions. This negative interaction between Syn6803 and Cf9212 under VL conditions can be classified as amensalism (0,-), where one organism is negatively affected while the other remains unaffected (17, 19). This negative interaction may also be classified as allelopathy in which one organism releases chemicals or compounds that inhibit the growth of another organism (20). However, because we do not have information on whether Syn6803 secretes any substances into the medium that affect Cf9212 in coculture, further studies are required to confirm the specific type of negative interaction between Syn6803 and Cf9212 under VL conditions. Furthermore, it is important to highlight that while we showed higher stress in Cf9212 under VL compared to FRL in the coculture, it should be noted that the intensities and exposure times of VL and FRL in our experimental setup were not identical. Hence, it was plausible that additional variables such as light intensity and duration of exposure could contribute to the observed negative interaction.

FRL-using and VL-using cyanobacteria coexist in the same habitats in the natural environments, including microbial mats in hot springs and beach rocks. Some FRL-using cyanobacteria reside in the deeper spaces of these microbial mats, whereas VL-using photoautotrophs, which include cyanobacteria, inhabit the upper depths (6, 15). Due to their shared capability of utilizing VL for photosynthesis, these two cyanobacteria compete for living space, nutrients, and VL in the upper layers of the microbial mats. This competitive interaction is consistent with the findings of our study, which demonstrates that Cf9212 underwent significant stress in VL when cultivated with Syn6803; metabolic transcript levels, cell density, and biomass were all reduced (Fig. 2B, 6E, and 7). However, FRL-using cyanobacteria do not face competition in the deep layers of the microbial mats, as VL-using cyanobacteria are incapable of utilizing FRL for photosynthesis, and FRL causes them extreme stress (Fig. 7; Fig. S3) (16). The advantage of FRL-using cyanobacteria to thrive in the deep layers is consistent with our findings that coculture minimally affects Cf9212 under FRL (Fig. 2B; Fig. S9). As a result, cyanobacteria that utilize FRL inhabit ecological niches at deep layers in the mats, where competition with other cyanobacteria is minimal.

### Conclusions

In summary, this study indicates that the growth of a VL-using cyanobacterium, Syn6803, was not impacted when cocultured in VL with a FRL-using cyanobacterium, Cf9212. On the contrary, the growth of Cf9212 was inhibited by Syn6803, as suggested by growth, pigment, and transcriptomic analyses. Remarkably, the inhibition was not from nutrient deprivation in coculture. The stress from Syn6803 to Cf9212 was weaker in FRL than in VL. Furthermore, Syn6803 was subjected to substantial stress under FRL conditions, and coculture further stressed it under FRL conditions. Our findings contribute to a better understanding of the potentially negative interaction between VL-using cyanobacteria and FRL-using cyanobacteria and the response of these two different species when they grow together.

## Data Availability

The raw reads of the transcriptomic data have been deposited in the NCBI under BioProject accession number PRJNA1076667.

## SUPPLEMENTAL MATERIAL

Supplemental information msphere.00251-24-s0001.docx Figures S1-S9; Tables S1 and S4-S9.

Table S2 msphere.00251-24-s0002.xlsx Normalized read counts from the transcriptomic data of Syn6803.

Table S3 msphere.00251-24-s0003.xlsx Normalized read counts from the transcriptomic data of Cf9212.

ASM does not own the copyrights to Supplemental Material that may be linked to, or accessed through, an article. The authors have granted ASM a non-exclusive, world-wide license to publish the Supplemental Material files. Please contact the corresponding author directly for reuse.
