# Supplementary material for: Two cyanobacterial species exhibit stress responses when grown together in visible light or far-red light
Source: mSphere. 2024 Aug 9;9(9):e00251-24. doi: 10.1128/msphere.00251-24 (PMC11423583; doi:10.1128/msphere.00251-24)
Supplement: Supplemental information — Figures S1-S9; Tables S1 and S4-S9. [file msphere.00251-24-s0001.docx]

**Supplementary Information**


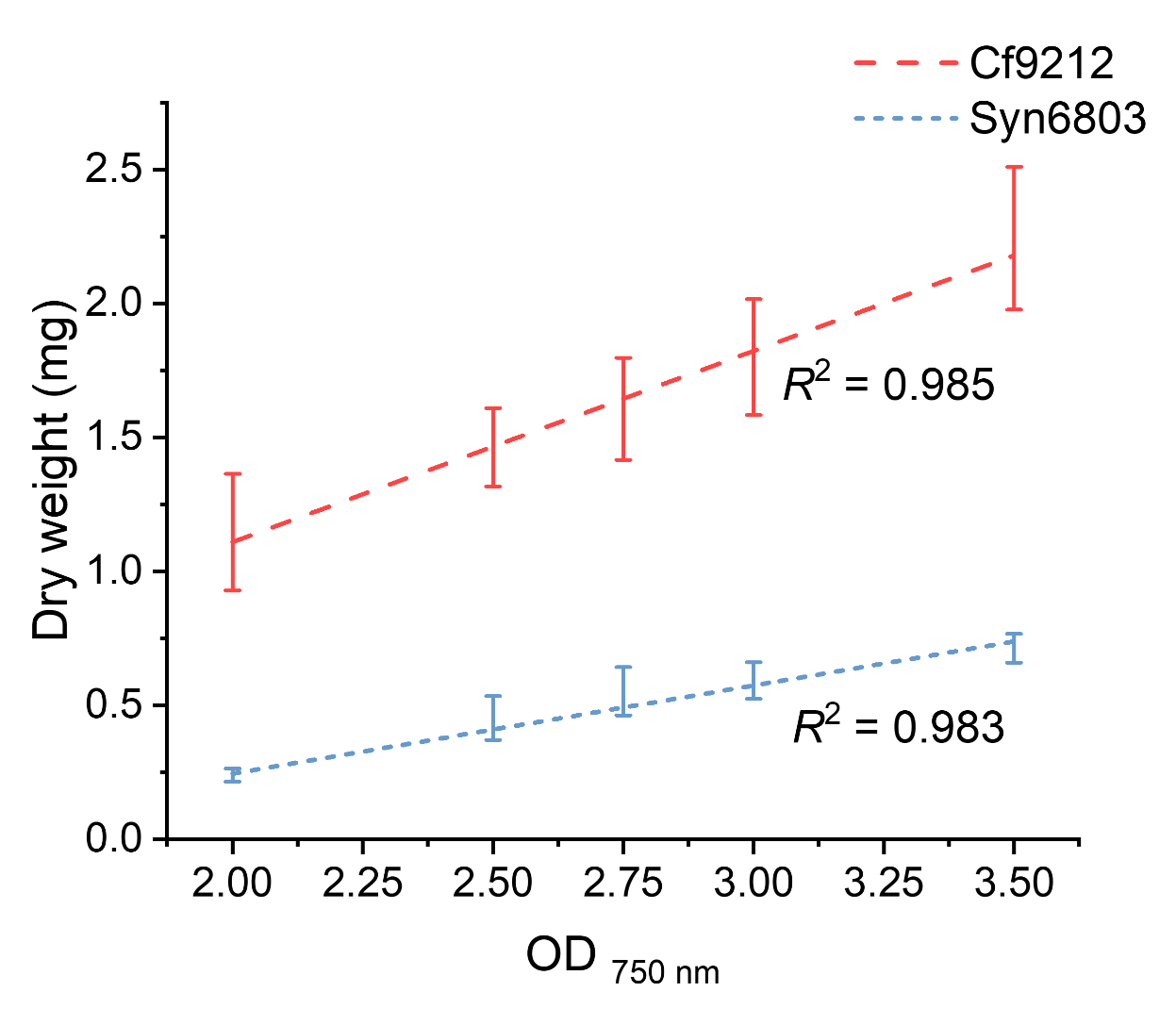


**Figure S1.** Calibration curves illustrate the linear relationship between the OD_750 nm_ and the corresponding dry weight of Syn6803 and Cf9212. The cyanobacterial cells in the exponential phase (OD_750 nm_ = 0.6 – 0.8) were concentrated for the measurement. The linear range of the calibration curves spans OD_750 nm_ values between 2.0 and 3.5 in 1 mL of the cell culture, and their goodness of fit can be calculated with an *R*^2^ > 0.98. Syn6803 and Cf9212 with an initial OD_750 nm_ of 0.025 in 100 mL volume were utilized for growth, pigment, and transcriptomic experiments.

**
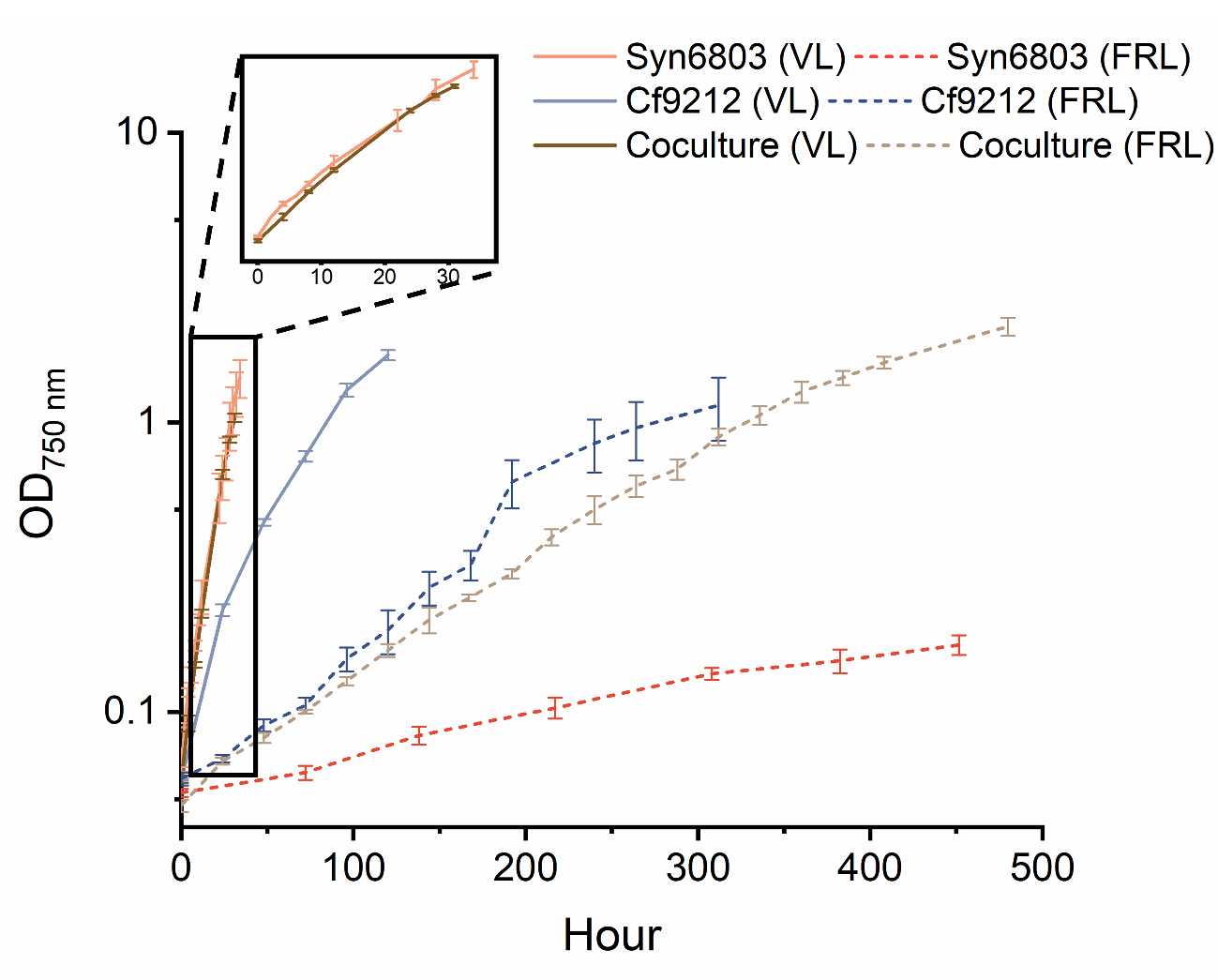
**

**Figure S2.** Growth curves of Syn6803 and Cf9212 monocultures and the coculture (Syn6803 + Cf9212) grown under VL and FRL. The y-axis is displayed on a logarithmic scale. Each point represents the mean of OD_750 nm_ ± standard deviation of three biological replicates. Doubling times were calculated during the exponential growth phase. The growth curves of Syn6803 monoculture are derived from Nien et al., 2022.

**
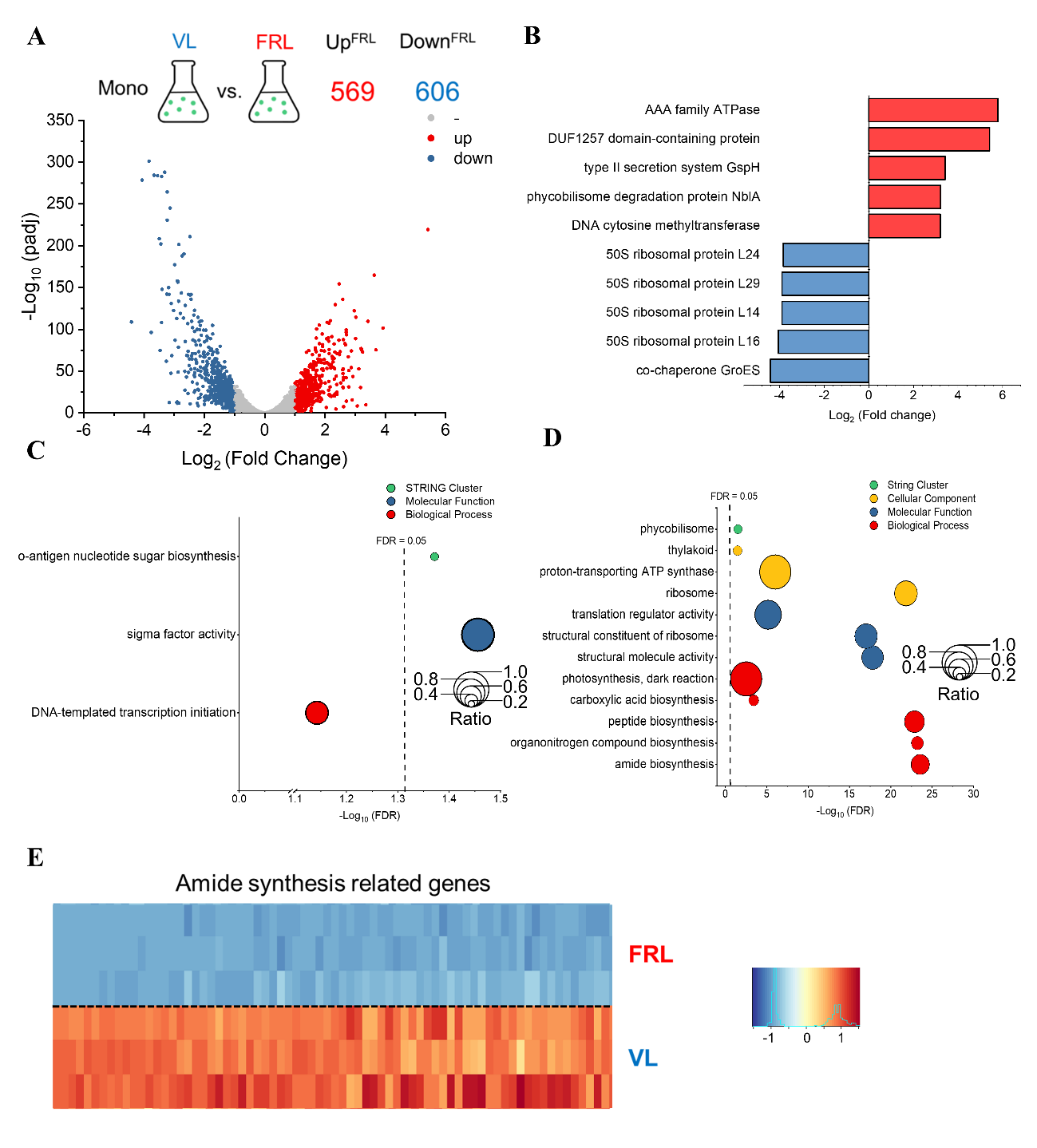
**

**Figure S3.** Transcriptomic profiles and gene ontology enrichment analysis performed on DEGs from Syn6803 monoculture. **(A)** Volcano plot comparing transcriptomic profiles of Syn6803 in far-red light (FRL) to visible light (VL). Genes with adjusted p values <0.05 and log2(fold change) >1 are highlighted in red (upregulated) and blue (downregulated). **(B)** Top 10 regulated DEGs from comparing Syn6803 monoculture between VL and FRL. Genes upregulated in FRL are marked in red, and genes downregulated in FRL are marked in blue. Bubble plots of function annotations from **(C)** upregulated genes and **(D)** downregulated genes in FRL. Each dot represents a function annotation from the GO database (red for biological process, blue for molecular function, yellow for cellular component) or STRING database (green). The size of the dots is defined as the ratio of DEGs to total genes in each function annotation. The vertical dashed line represents a false discovery rate of 0.05. **(E)** Heatmap of amide biosynthesis-related genes. The color scale represents the z-score values of the gene expression levels, with red indicating upregulation and blue indicating downregulation in FRL compared to VL.


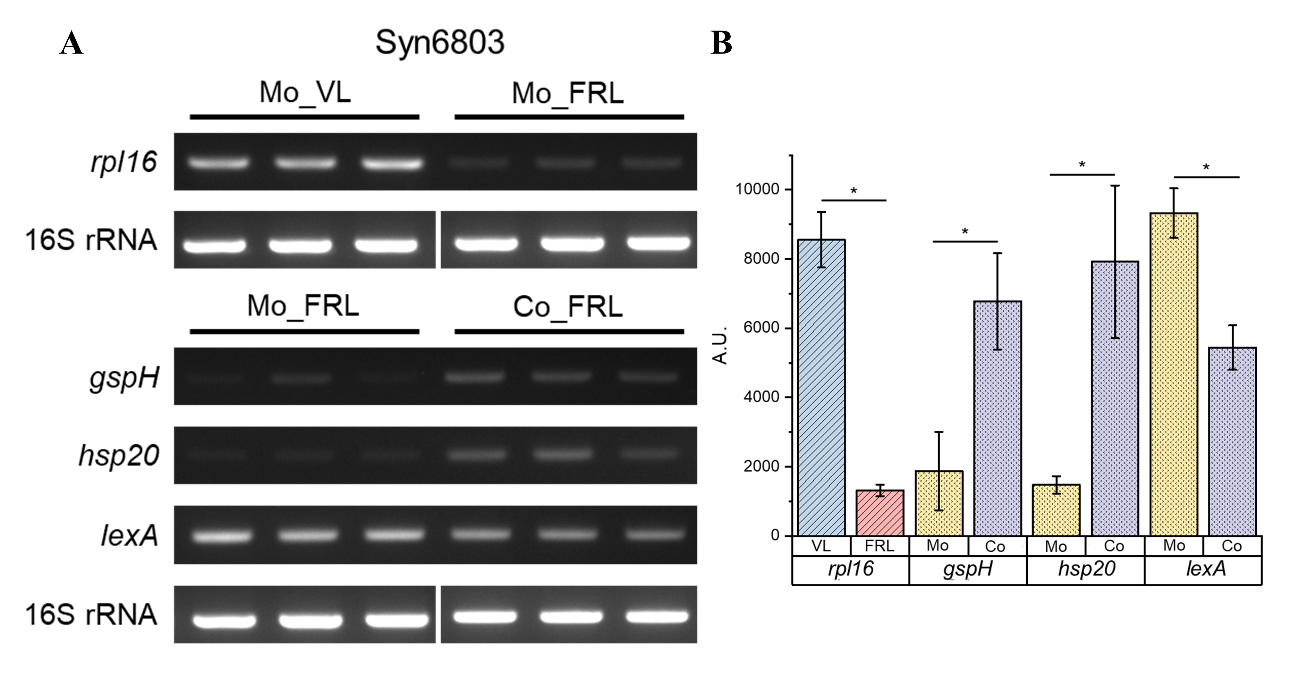


**Figure S4.** RT-PCR analysis of selected genes from Syn6803. **(A)** Electrophoretic analysis of amplicons from RT-PCR analysis. Four genes, namely *rpl16*, *gspH*, *hsp20*, and *lexA*, along with the housekeeping gene 16S rRNA, were selected for RT-PCR. **(B)** Quantification of the relative expression levels of the selected genes in panel A. The relative expression level of *rpl16* is compared between Syn6803 monoculture under VL and FRL, while the relative expression level of *gspH*, *hsp20*, and *lexA* are compared between Syn6803 monoculture and coculture under FRL. Mann-Whitney U test was conducted based on three replicates. Statistical significance levels are indicated as follows: *P<0.05; ns, non-significant.


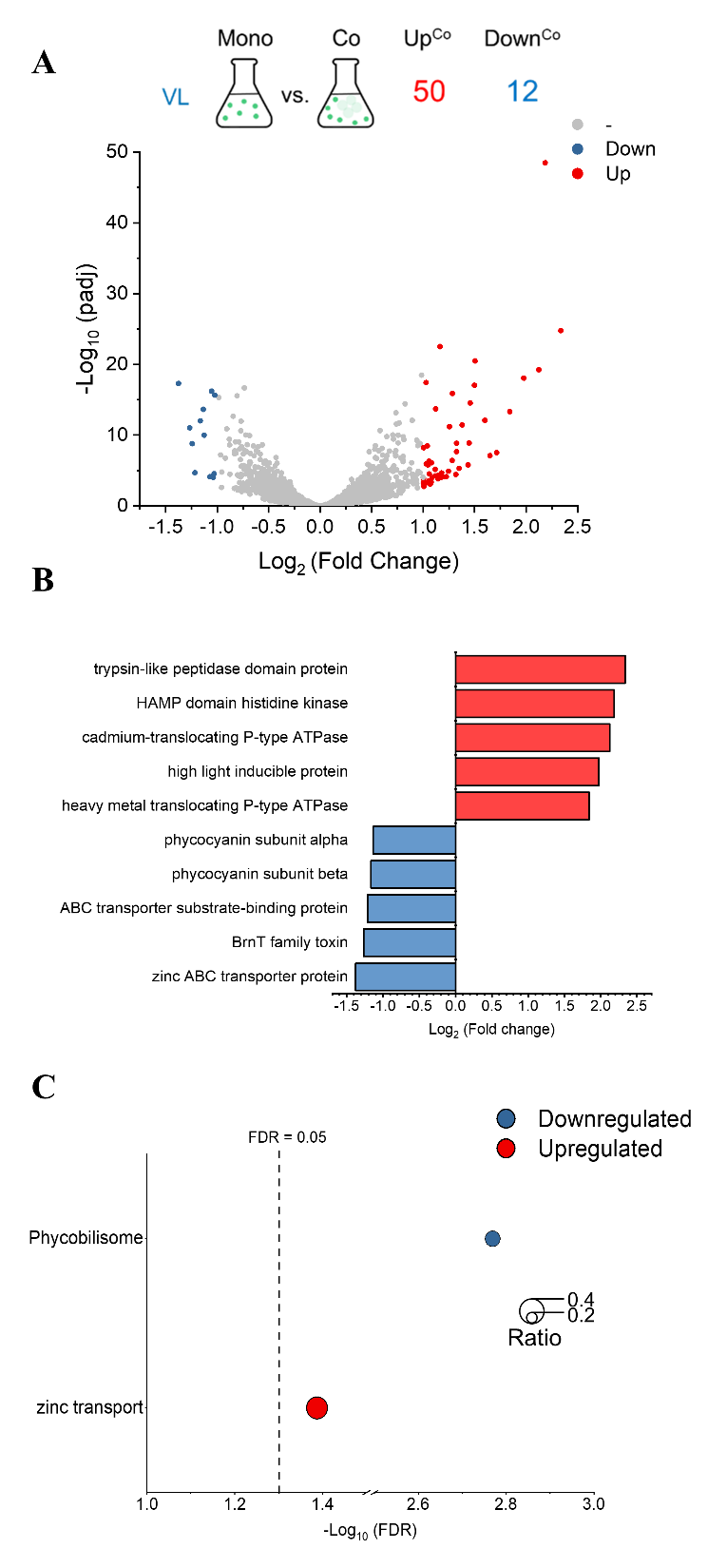


**Figure S5.** Transcriptomic profiles and gene ontology enrichment analysis of DEGs from the comparison between monoculture and coculture of Syn6803 in VL. **(A)** Volcano plot of Syn6803 transcriptomic profiles in monoculture and coculture under VL. Genes with adjusted p values <0.05 and log2(fold change) >1 are highlighted in red (upregulated) and blue (downregulated). **(B)** Top 10 regulated DEGs from the comparison between monoculture and coculture of Syn6803 in VL. Genes upregulated in coculture are marked in red, and genes downregulated in coculture are marked in blue. **(C)** Bubble plot of function annotations from upregulated and downregulated genes in coculture. The dots represent function annotations from the STRING database. The size of the dots is defined as the ratio of DEGs to total genes in each function annotation. The vertical dashed line represents a false discovery rate of 0.05.


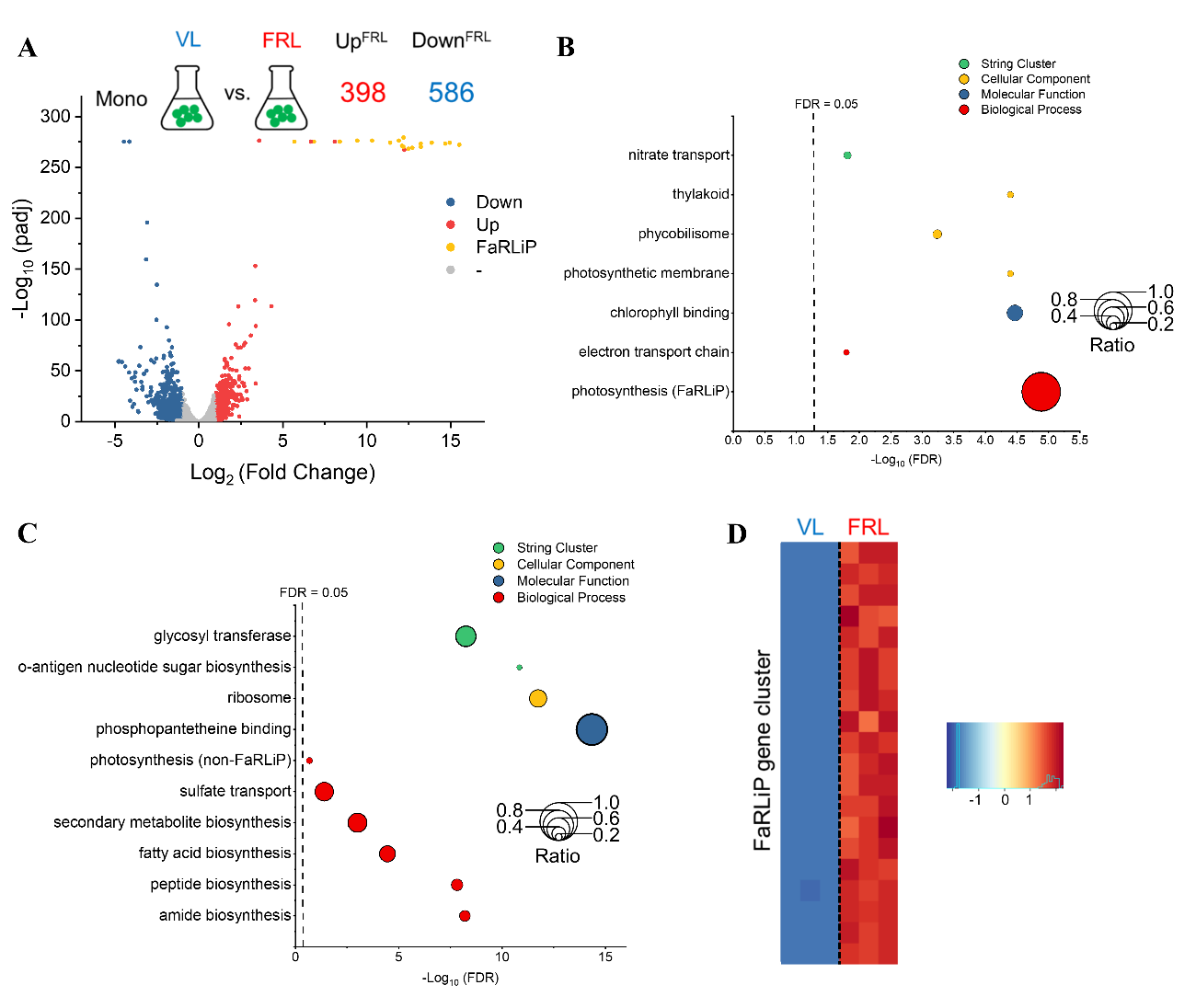


**Figure S6**. Transcriptomic profiles and gene ontology enrichment analysis of DEGs from Cf9212 monoculture. **(A)** Volcano plot comparing transcriptomic profiles of Cf9212 in far-red light (FRL) to visible light (VL). Genes with adjusted p values <0.05 and log2(fold change) >1 are highlighted in red (upregulation) and blue (downregulation). The FaRLiP gene cluster is highlighted in orange. Bubble plot of function annotations from **(B)** upregulated genes and **(C)** downregulated genes in FRL. Each dot represents a function annotation from the GO database (red for biological process, blue for molecular function, yellow for cellular component) or STRING database (green). The size of the dots is defined as the ratio of DEGs to total genes in each function annotation. The vertical dashed line represents a false discovery rate of 0.05. **(D)** Heatmap of the FaRLiP gene cluster identified in panel B. The color scale represents the z-score values of the gene expression levels, with red indicating upregulation and blue indicating downregulation in FRL compared to VL.


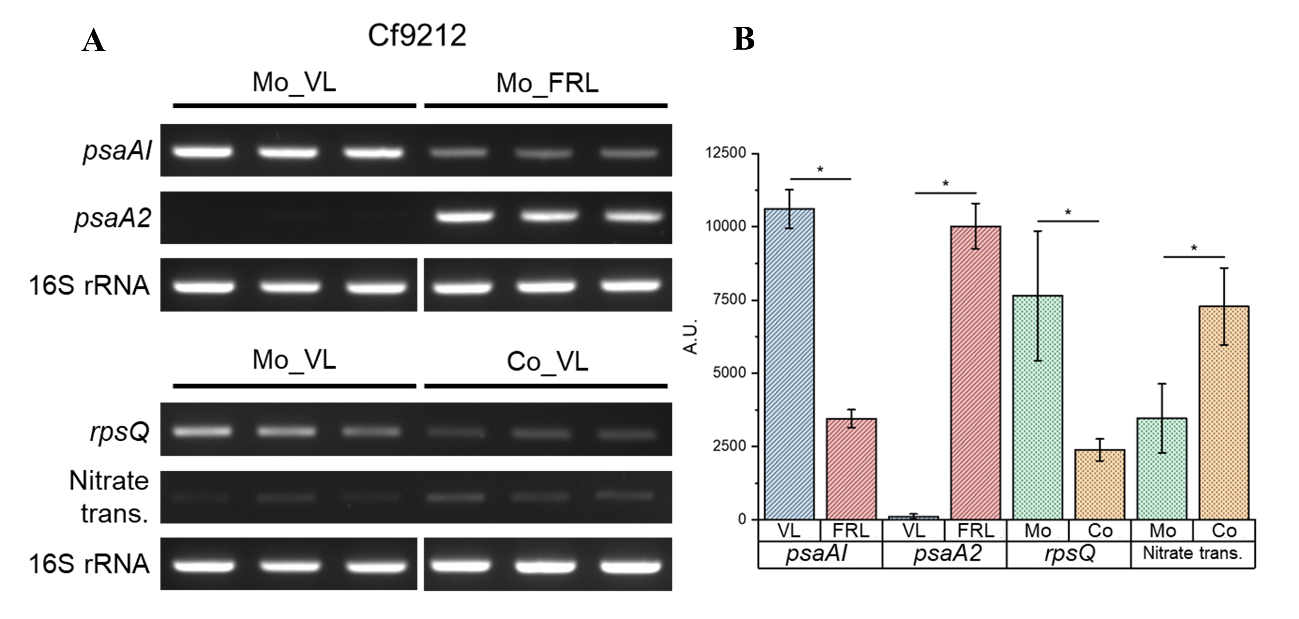


**Figure S7.** RT-PCR analysis of selected genes from Cf9212. **(A)** Electrophoretic analysis of amplicons from RT-PCR analysis. Four genes, namely *psaA1*, *psaA2*, *rpsQ*, and a gene encoding nitrate transporter (nitrate trans.), along with the housekeeping gene 16S rRNA, were selected for RT-PCR. **(B)** Quantification of the relative expression levels of the selected genes in panel A. The relative expression levels of *psaA1* and *psaA2* are compared between Cf9212 monoculture under VL and FRL, while the relative expression level of *rpsQ* and nitrate trans. are compared between Cf9212 monoculture and coculture under VL. Mann-Whitney U test was conducted based on three replicates. Statistical significance levels are indicated as follows: *P<0.05; ns, non-significant.


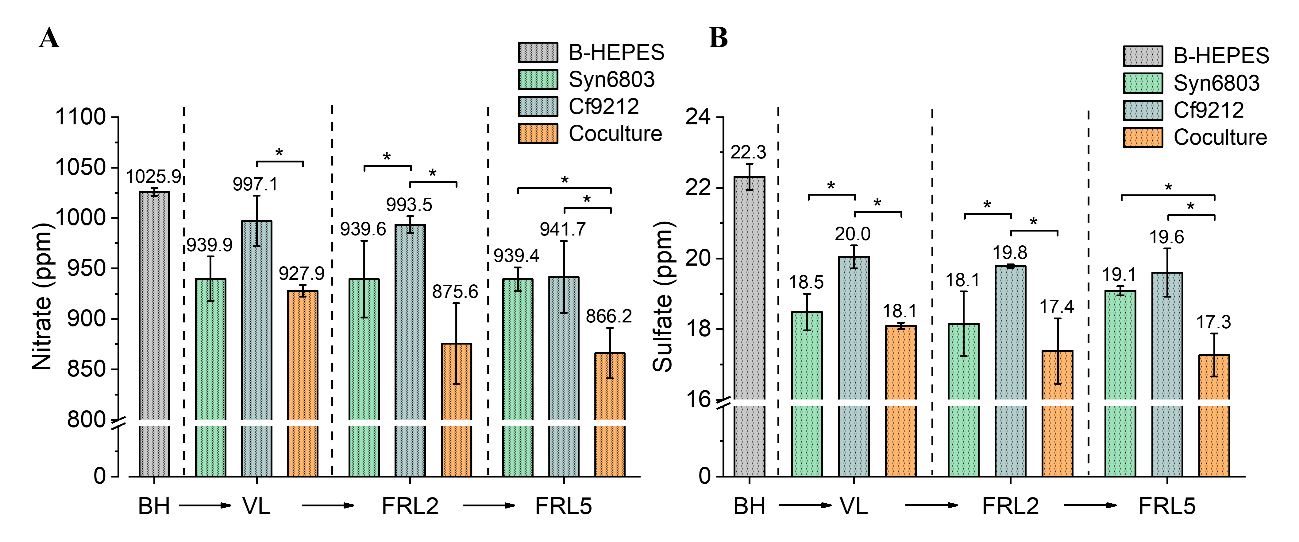


**Figure S8.** The measurement of **(A)** nitrate and **(B)** sulfate concentration in Syn6803 monoculture, Cf9212 monoculture, and coculture cell medium after growing under VL for 30 hours (VL), after transferring from VL to FRL for 2 days (FRL2) and 5 days (FRL5), respectively. Nitrate and sulfate concentrations in fresh B-HEPES (BH) medium were also compared. Concentrations are reported in parts per million (ppm). Kruskal-Wallis tests, followed by Mann-Whitney U tests, were conducted based on three replicates. Statistical significance levels are indicated as follows: *P<0.05.


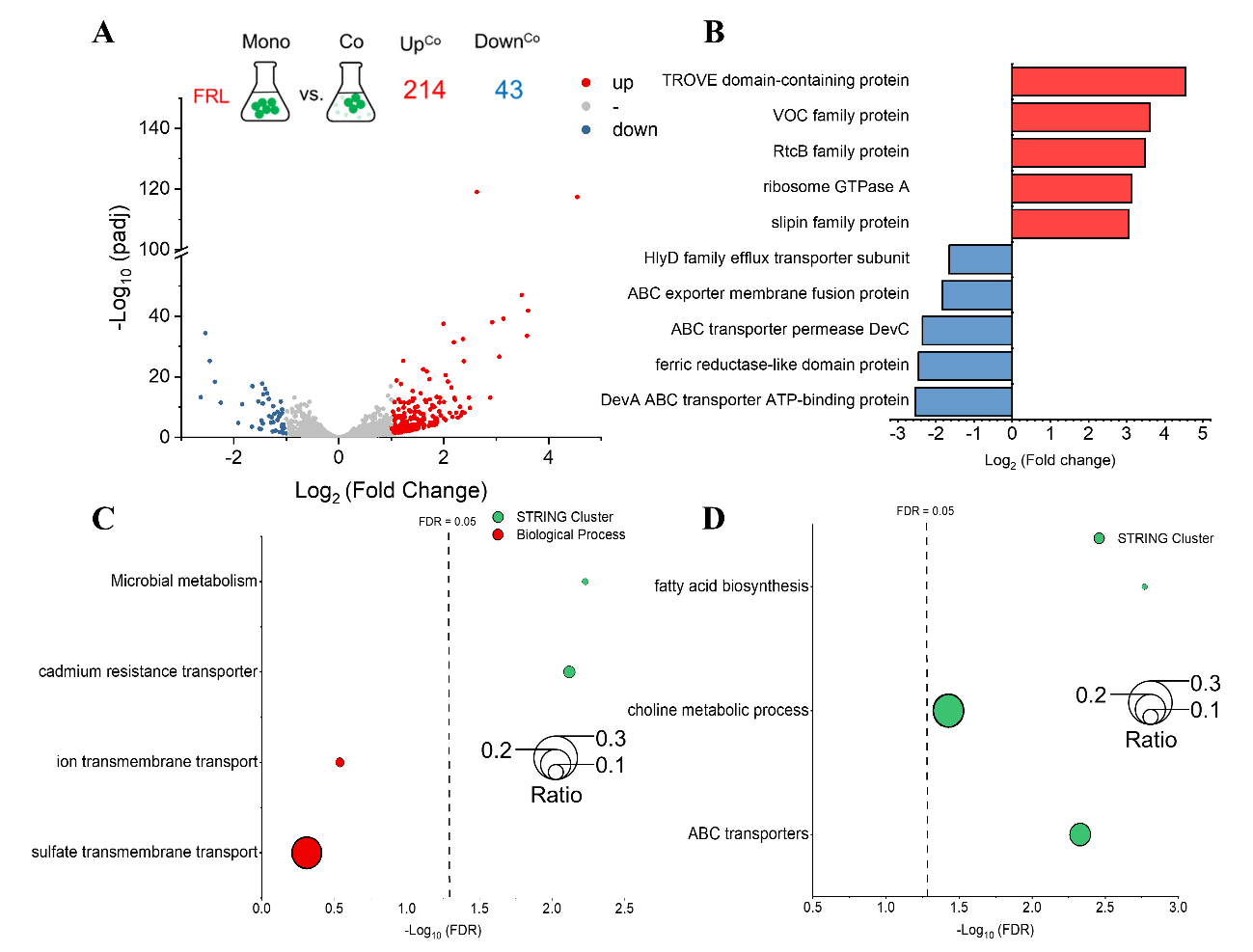


**Figure S9**. Transcriptomic profiles and gene ontology enrichment analysis of DEGs from the comparison between monoculture and coculture of Cf9212 in FRL. **(A)** Volcano plot of Cf9212 transcriptomic profiles in monoculture and coculture under FRL. Genes with adjusted p values <0.05 and log2(fold change) >1 are highlighted in red (upregulated) and blue (downregulated). **(B)** Top 10 regulated DEGs from the comparison between monoculture and coculture of Cf9212 in FRL. Genes upregulated in coculture are marked in red, and genes downregulated in coculture are marked in blue. Bubble plots of function annotations from **(C)** upregulated genes and **(D)** downregulated genes in coculture. Each dot represents a function annotation from the GO database (red for the biological process) or STRING database (green). The size of the dots is defined as the ratio of DEGs to total genes in each function annotation. The vertical dashed line represents a false discovery rate of 0.05.

**Table S1.** Primers used in this study

| Primer name | Length | Sequence (5’ – 3’) | Description |
| --- | --- | --- | --- |
| TNrt03 16s_F | 20 | GAGAGTTTGATCCTGGCTCA | Amplification of ~200-bp region in 16s rRNA |
| TNrt04 16s_R | 20 | ATCTAGCTAATCAGACGCGA | Amplification of ~200-bp region in 16s rRNA |
| TNrt05 psaA2_9212_F | 20 | TCAGTTCTTGCCAGTAGCCG | Amplification of ~200-bp region in *psaA2* in Cf9212 |
| TNrt06 psaA2_9212_R | 21 | GGTGGAAACTTTGCGACTTCG | Amplification of ~200-bp region in *psaA2* in Cf9212 |
| TNrt07 psaA1_9212_F | 20 | GTGGTATCCTCGCTGTAGGC | Amplification of ~200-bp region in *psaA1* in Cf9212 |
| TNrt08 psaA1_9212_R | 20 | GCCCAAGTTTGCCTTGTCTG | Amplification of ~200-bp region in *psaA1* in Cf9212 |
| TNrt09 50S_ribo_6803_R | 22 | ACCGAACCCTGTTGGATCACCT2 | Amplification of ~200-bp region in *rpl16* in Syn6803 |
| TNrt10 50S_ribo_6803_F | 22 | CGGGCTTAACTACCGCTACCCA | Amplification of ~200-bp region in *rpl16* in Syn6803 |
| TNrt11 Type II sec_6803_R | 22 | TCTTTCGCTGAGAGCAACCAAG | Amplification of ~200-bp region in *gspH* in Syn6803 |
| TNrt12 Type II sec_6803_F | 21 | CTCGGCTGGAACAGGACTTTG | Amplification of ~200-bp region in *gspH* in Syn6803 |
| TNrt13 LexA_6803_R | 22 | TCACCCGAGCCCAAAAAGAACT | Amplification of ~200-bp region in *lexA* in Syn6803 |
| TNrt14 LexA_6803_F | 22 | GGCTTTGCCGTCAGTCCAATCA2 | Amplification of ~200-bp region in *lexA* in Syn6803 |
| TNrt15 Hsp20_6803_R | 22 | TCGCCAGGATACCCATAGCACC2 | Amplification of ~200-bp region in *hsp20* in Syn6803 |
| TNrt16 Hsp20_6803_F | 24 | TTGGCTTCCTCTACTTTGGGCAAA | Amplification of ~200-bp region in *hsp20* in Syn6803 |
| TNrt17 30s_ribo_9212_F | 21 | TGGTAGTCGCCGTTGAAAACC | Amplification of ~200-bp region in *rpsQ* in Cf9212 |
| TNrt18 30s_ribo_9212_R | 22 | AGGATTTCTGTGACTTGCCAGC | Amplification of ~200-bp region in *rpsQ* in Cf9212 |
| TNrt19 Nit_transport_9212_F | 20 | TCGCCAAGCACGGTTTAGAT | Amplification of ~200-bp region in the nitrate transporter-related gene in Cf9212 |
| TNrt20 Nit_transport_9212_R | 20 | CGGCGACAGTTTGTATCCCT | Amplification of ~200-bp region in the nitrate transporter-related gene in Cf9212 |

**Table S2.** Normalized read counts from the transcriptomic data of Syn6803. (uploaded as a separate file)

**Table S3.** Normalized read counts from the transcriptomic data of Cf9212. (uploaded as a separate file)

**Table S4.** Average transcript levels of the genes related to amide biosynthesis from the transcriptomic data of Syn6803 monoculture under VL and FRL. The transcript levels of each gene are normalized as TPM. The average transcript levels were calculated based on three replicates. Only the DEGs are shown in the Table. The locus tag of each gene was assigned based on the annotations in CyanoOmicsDB (http://www.cyanoomics.cn)

| **Amide biosynthesis (Syn6803_Mo_VL vs. Mo_FRL)** | | | | | |
| --- | --- | --- | --- | --- | --- |
| **Locus tag** | **Product** | **Mo_VL** | **Mo**  **FRL** | **log2Fold Change** | **adjusted p-value** |
| SGL_RS00545 | aminoacyl-tRNA hydrolase | 46.61 | 19.30 | -1.15 | <0.001 |
| SGL_RS03445 | 30S ribosomal protein S15 | 1177.60 | 514.82 | -1.07 | <0.001 |
| SGL_RS03485 | translation initiation factor IF-3 | 620.38 | 129.66 | -2.13 | <0.001 |
| SGL_RS04920 | tyrosine--tRNA ligase | 62.47 | 17.17 | -1.74 | <0.001 |
| SGL_RS05225 | serine--tRNA ligase | 124.95 | 46.99 | -1.28 | <0.001 |
| SGL_RS05710 | 50S ribosomal protein L31 | 1039.62 | 239.11 | -1.98 | <0.001 |
| SGL_RS05715 | 30S ribosomal protein S9 | 1299.49 | 294.61 | -2.02 | <0.001 |
| SGL_RS05720 | 50S ribosomal protein L13 | 1126.90 | 243.00 | -2.09 | <0.001 |
| SGL_RS05730 | 50S ribosomal protein L17 | 1449.23 | 245.37 | -2.44 | <0.001 |
| SGL_RS05740 | 30S ribosomal protein S11 | 1142.09 | 181.34 | -2.53 | <0.001 |
| SGL_RS05745 | 30S ribosomal protein S13 | 1462.70 | 337.37 | -1.99 | <0.001 |
| SGL_RS05750 | translation initiation factor IF-1 | 226.60 | 99.38 | -1.07 | <0.001 |
| SGL_RS05765 | 50S ribosomal protein L15 | 1004.71 | 138.33 | -2.73 | <0.001 |
| SGL_RS05770 | 30S ribosomal protein S5 | 1640.06 | 269.79 | -2.48 | <0.001 |
| SGL_RS05775 | 50S ribosomal protein L18 | 1690.68 | 241.97 | -2.68 | <0.001 |
| SGL_RS05780 | 50S ribosomal protein L6 | 1737.09 | 169.31 | -3.23 | <0.001 |
| SGL_RS05785 | 30S ribosomal protein S8 | 1642.25 | 127.74 | -3.56 | <0.001 |
| SGL_RS05790 | 50S ribosomal protein L5 | 1737.67 | 111.19 | -3.83 | <0.001 |
| SGL_RS05795 | 50S ribosomal protein L24 | 2030.51 | 129.74 | -3.84 | <0.001 |
| SGL_RS05800 | 50S ribosomal protein L14 | 1726.19 | 105.83 | -3.90 | <0.001 |
| SGL_RS05805 | 30S ribosomal protein S17 | 1894.21 | 135.14 | -3.68 | <0.001 |
| SGL_RS05810 | 50S ribosomal protein L29 | 2172.85 | 133.38 | -3.90 | <0.001 |
| SGL_RS05815 | 50S ribosomal protein L16 | 1774.79 | 96.26 | -4.07 | <0.001 |
| SGL_RS05820 | 30S ribosomal protein S3 | 1546.86 | 150.46 | -3.23 | <0.001 |
| SGL_RS05825 | 50S ribosomal protein L22 | 1864.95 | 156.03 | -3.44 | <0.001 |
| SGL_RS05830 | 30S ribosomal protein S19 | 1620.75 | 131.13 | -3.49 | <0.001 |
| SGL_RS05835 | 50S ribosomal protein L2 | 1222.79 | 104.75 | -3.41 | <0.001 |
| SGL_RS05840 | 50S ribosomal protein L23 | 1597.94 | 184.27 | -2.97 | <0.001 |
| SGL_RS05845 | 50S ribosomal protein L4 | 1007.94 | 132.05 | -2.80 | <0.001 |
| SGL_RS05850 | 50S ribosomal protein L3 | 927.39 | 135.31 | -2.64 | <0.001 |
| SGL_RS06195 | 50S ribosomal protein L7/L12 | 2097.79 | 259.68 | -2.88 | <0.001 |
| SGL_RS06200 | 50S ribosomal protein L10 | 1725.69 | 179.53 | -3.13 | <0.001 |
| SGL_RS06205 | 50S ribosomal protein L1 | 1363.08 | 287.47 | -2.12 | <0.001 |
| SGL_RS06210 | 50S ribosomal protein L11 | 1040.27 | 280.37 | -1.77 | <0.001 |
| SGL_RS06345 | alpha-ketoacid dehydrogenase subunit beta | 127.97 | 52.85 | -1.15 | <0.001 |
| SGL_RS06545 | lysine--tRNA ligase | 99.47 | 28.90 | -1.66 | <0.001 |
| SGL_RS06700 | peptide chain release factor 3 | 191.39 | 49.20 | -1.84 | <0.001 |
| SGL_RS06865 | isoleucine--tRNA ligase | 91.68 | 29.23 | -1.53 | <0.001 |
| SGL_RS07160 | amidotransferase subunit GatA | 260.03 | 72.69 | -1.72 | <0.001 |
| SGL_RS07595 | 30S ribosomal protein S6 | 912.83 | 329.90 | -1.35 | <0.001 |
| SGL_RS08945 | bifunctional phosphopantothenoylcysteine decarboxylase/phosphopantothenate--cysteine ligase CoaBC | 67.31 | 22.17 | -1.48 | <0.001 |
| SGL_RS10000 | translation elongation factor Ts | 399.41 | 85.18 | -2.10 | <0.001 |
| SGL_RS10005 | 30S ribosomal protein S2 | 705.84 | 187.13 | -1.79 | <0.001 |
| SGL_RS10920 | 30S ribosomal protein S10 | 779.95 | 177.12 | -2.01 | <0.001 |
| SGL_RS10925 | elongation factor Tu | 2885.36 | 801.97 | -1.73 | <0.001 |
| SGL_RS10930 | elongation factor G | 882.28 | 377.04 | -1.11 | <0.001 |
| SGL_RS10935 | 30S ribosomal protein S7 | 945.92 | 353.63 | -1.30 | <0.001 |
| SGL_RS11940 | hypothetical protein | 3201.01 | 1327.89 | -1.15 | <0.001 |
| SGL_RS11990 | alanine--tRNA ligase | 59.78 | 22.89 | -1.26 | <0.001 |
| SGL_RS12170 | ribosome recycling factor | 296.26 | 117.44 | -1.21 | <0.001 |
| SGL_RS12270 | peptide chain release factor 2 | 57.40 | 16.92 | -1.63 | <0.001 |
| SGL_RS12345 | pyruvate dehydrogenase | 102.01 | 39.60 | -1.23 | <0.001 |
| SGL_RS13065 | 50S ribosomal protein L20 | 1663.54 | 757.48 | -1.01 | <0.001 |
| SGL_RS13565 | glycine--tRNA ligase subunit beta | 87.77 | 30.48 | -1.40 | <0.001 |
| SGL_RS13730 | elongation factor P | 333.51 | 125.59 | -1.28 | <0.001 |
| SGL_RS14010 | 30S ribosomal protein S4 | 641.88 | 270.77 | -1.13 | <0.001 |
| SGL_RS14135 | phenylalanine--tRNA ligase subunit alpha | 129.67 | 39.78 | -1.57 | <0.001 |
| SGL_RS14465 | acyl-ACP--UDP-N-acetylglucosamine O-acyltransferase | 69.88 | 29.79 | -1.11 | <0.001 |
| SGL_RS14545 | GTP cyclohydrolase I FolE | 241.01 | 85.77 | -1.36 | <0.001 |
| SGL_RS14905 | 30S ribosomal protein PSRP-3 | 733.02 | 267.66 | -1.33 | <0.001 |
| SGL_RS14975 | translation initiation factor | 338.65 | 109.69 | -1.50 | <0.001 |
| SGL_RS15030 | acetate--CoA ligase | 232.96 | 73.20 | -1.55 | <0.001 |
| SGL_RS15080 | 30S ribosomal protein S18 | 517.49 | 140.59 | -1.75 | <0.001 |
| SGL_RS16510 | branched-chain amino acid transaminase | 351.38 | 99.75 | -1.69 | <0.001 |
| SGL_RS16615 | 3-methyl-2-oxobutanoate hydroxymethyltransferase | 64.64 | 28.75 | -1.05 | <0.001 |
| SGL_RS16775 | asparagine--tRNA ligase | 45.49 | 19.78 | -1.08 | <0.001 |
| SGL_RS16940 | dephospho-CoA kinase | 74.43 | 13.76 | -2.31 | <0.001 |
| SGL_RS16980 | valine--tRNA ligase | 123.53 | 42.89 | -1.41 | <0.001 |
| SGL_RS17215 | ketol-acid reductoisomerase | 451.81 | 185.21 | -1.17 | <0.001 |
| SGL_RS18015 | dihydroxy-acid dehydratase | 635.08 | 142.80 | -2.03 | <0.001 |
| SGL_RS18110 | peptide chain release factor 1 | 80.78 | 28.49 | -1.38 | <0.001 |
| SGL_RS18715 | 50S ribosomal protein L36 | 2098.04 | 885.52 | -1.12 | <0.001 |
| SGL_RS19055 | 50S ribosomal protein L33 | 599.05 | 154.09 | -1.82 | <0.001 |

**Table S5.** Average transcript levels of the genes related to type II secretion system from the transcriptomic data of Syn6803 monoculture and coculture under FRL. The transcript levels of each gene are normalized as TPM. The average transcript levels were calculated based on three replicates. Only the DEGs are shown in the Table. The locus tag of each gene was assigned based on the annotations in CyanoOmicsDB (http://www.cyanoomics.cn)

| **Type II secretion system (Syn6803_Mo_FRL vs. Co_FRL)** | | | | | |
| --- | --- | --- | --- | --- | --- |
| **Gene**  **Symbol** | **Product** | **Mo**  **FRL** | **Co**  **FRL** | **log2Fold Change** | **adjusted p-value** |
| SGL_RS07940 | type II secretion system GspH family protein | 229.72 | 1337.74 | 1.95 | <0.001 |
| SGL_RS07935 | hypothetical protein | 26.00 | 135.53 | 1.80 | <0.001 |
| SGL_RS17165 | type II secretion system protein GspG | 79.80 | 347.03 | 1.63 | <0.001 |
| SGL_RS07945 | prepilin-type N-terminal cleavage/methylation domain-containing protein | 8077.32 | 28528.48 | 1.52 | <0.001 |
| SGL_RS07950 | class I SAM-dependent methyltransferase | 27.83 | 80.37 | 1.39 | <0.001 |
| SGL_RS15295 | GspE/PulE family protein | 109.03 | 287.90 | 1.17 | <0.001 |

**Table S6.** Average transcript levels of the FaRLiP gene cluster from the transcriptomic data of Cf9212 monoculture under VL and FRL. The transcript levels of each gene are normalized as TPM. The average transcript levels were calculated based on three replicates. The locus tag of each gene was assigned based on the annotations in CyanoOmicsDB (http://www.cyanoomics.cn)

| **FaRLiP gene cluster (Cf9212 Mo_VL vs. Mo_FRL)** | | | | | |
| --- | --- | --- | --- | --- | --- |
| **Gene**  **Symbol** | **Product** | **Mo_VL** | **Mo_FRL** | **log2Fold Change** | **adjusted p-value** |
| UYE_RS0105845 | ApcB2 | 1.73 | 15416.23 | 13.22 | <0.001 |
| UYE_RS0105850 | ApcD2 | 0.78 | 5438.26 | 12.72 | <0.001 |
| UYE_RS0105860 | ApcD3 | 1.79 | 9490.15 | 12.49 | <0.001 |
| UYE_RS0105840 | ApcD5 | 2.90 | 25223.24 | 13.22 | <0.001 |
| UYE_RS0105855 | ApcE2 | 1.43 | 6022.39 | 12.19 | <0.001 |
| UYE_RS0105795 | PsaA2 | 0.62 | 17974.64 | 14.95 | <0.001 |
| UYE_RS0105790 | PsaB2 | 0.37 | 16756.03 | 15.52 | <0.001 |
| UYE_RS0105890 | PsaF2 | 2.98 | 3461.26 | 10.32 | <0.001 |
| UYE_RS34630 | PsaI2 | 6.70 | 2081.50 | 8.40 | <0.001 |
| UYE_RS0105895 | PsaJ2 | 6.08 | 3903.45 | 9.43 | <0.001 |
| UYE_RS0105785 | PsaL2 | 3.66 | 1163.53 | 8.39 | <0.001 |
| UYE_RS0105835 | PsbA3 | 1.78 | 24390.39 | 13.87 | <0.001 |
| UYE_RS0105830 | PsbA4 | 0.67 | 17674.22 | 14.67 | <0.001 |
| UYE_RS0105875 | PsbB2 | 1.95 | 7868.17 | 12.10 | <0.001 |
| UYE_RS0105870 | PsbC2 | 2.43 | 10409.02 | 12.18 | <0.001 |
| UYE_RS0105865 | PsbD3 | 2.57 | 9776.86 | 11.91 | <0.001 |
| UYE_RS0105880 | PsbH2 | 3.13 | 7902.09 | 11.40 | <0.001 |
| UYE_RS0105820 | RfpA | 3.44 | 333.38 | 6.79 | <0.001 |
| UYE_RS0105825 | RfpB | 3.69 | 370.11 | 6.85 | <0.001 |
| UYE_RS0105815 | RfpC | 17.27 | 759.99 | 5.68 | <0.001 |

**Table S7.** Average transcript levels of the genes related to ion transporter from the transcriptomic data of Cf9212 monoculture and coculture under VL. The transcript levels of each gene are normalized as TPM. The average transcript levels were calculated based on three replicates. Only the DEGs are shown in the Table. The locus tag of each gene was assigned based on the annotations in CyanoOmicsDB (http://www.cyanoomics.cn)

| **Ion transporter (Cf9212_Mo_VL vs. Co_VL)** | | | | | |
| --- | --- | --- | --- | --- | --- |
| **Gene**  **Symbol** | **Product** | **Mo**  **VL** | **Co**  **VL** | **log2Fold Change** | **adjusted p-value** |
| UYE_RS0103115 | SulP family inorganic anion transporter | 0.56 | 1.86 | 1.24 | 0.022 |
| UYE_RS0103125 | cation transporter | 1.09 | 3.86 | 1.26 | 0.026 |
| UYE_RS0103190 | calcium/proton exchanger | 0.74 | 2.27 | 1.22 | 0.007 |
| UYE_RS0104880 | ABC transporter substrate-binding protein | 12.39 | 105.56 | 2.50 | <0.001 |
| UYE_RS0104885 | nitrate ABC transporter permease | 22.42 | 169.40 | 2.40 | <0.001 |
| UYE_RS0104890 | nitrate ABC transporter ATP-binding protein | 22.77 | 162.44 | 2.30 | <0.001 |
| UYE_RS0104895 | nitrate ABC transporter ATP-binding protein | 27.87 | 169.15 | 2.09 | <0.001 |
| UYE_RS0113535 | mechanosensitive ion channel family protein | 0.73 | 2.41 | 1.27 | 0.0079 |
| UYE_RS0114730 | sulfate/molybdate ABC transporter ATP-binding protein | 5.75 | 178.13 | 1.48 | 0.0088 |
| UYE_RS0114735 | sulfate ABC transporter substrate-binding protein | 6.42 | 254.90 | 1.72 | 0.0017 |
| UYE_RS0119035 | sulfate ABC transporter permease subunit CysW | 54.76 | 244.74 | 1.56 | 0.0019 |
| UYE_RS0119040 | sulfate ABC transporter permease subunit CysT | 70.79 | 298.46 | 1.38 | 0.021 |
| UYE_RS0123250 | sodium-dependent bicarbonate transport family permease | 86.91 | 337.13 | 1.44 | 0.0024 |
| UYE_RS0127355 | cation diffusion facilitator family transporter | 6.24 | 17.74 | 1.18 | <0.001 |
| UYE_RS0127370 | CusA/CzcA family heavy metal efflux RND transporter | 5.17 | 24.28 | 1.77 | <0.001 |
| UYE_RS0127860 | heavy metal translocating P-type ATPase | 0.56 | 3.11 | 1.81 | <0.001 |
| UYE_RS0128710 | magnesium transporter | 26.08 | 94.88 | 1.29 | 0.016 |
| UYE_RS0134020 | SulP family inorganic anion transporter | 88.89 | 500.38 | 1.73 | 0.0058 |
| UYE_RS0134025 | cation:proton antiporter | 71.12 | 345.11 | 1.59 | 0.013 |

**Table S8.** Average transcript levels of the genes related to ribosomal protein from the transcriptomic data of Cf9212 monoculture and coculture under VL. The transcript levels of each gene are normalized as TPM. The average transcript levels were calculated based on three replicates. Only the DEGs are shown in the Table. The locus tag of each gene was assigned based on the annotations in CyanoOmicsDB (http://www.cyanoomics.cn)

| **Ribosome (Cf9212_Mo_VL vs. Co_VL)** | | | | | |
| --- | --- | --- | --- | --- | --- |
| **Gene**  **Symbol** | **Product** | **Mo**  **VL** | **Co**  **VL** | **log2Fold Change** | **adjusted p-value** |
| UYE_RS0105520 | 30S ribosomal protein S4 | 584.94 | 318.14 | -1.03 | <0.001 |
| UYE_RS0116340 | 50S ribosomal protein L10 | 2014.77 | 961.48 | -1.26 | <0.001 |
| UYE_RS0116345 | 50S ribosomal protein L7/L12 | 1871.37 | 948.42 | -1.16 | <0.001 |
| UYE_RS0128465 | 50S ribosomal protein L3 | 1734.12 | 816.65 | -1.24 | <0.001 |
| UYE_RS0128475 | 50S ribosomal protein L23 | 2046.87 | 1012.92 | -1.20 | <0.001 |
| UYE_RS0128485 | 30S ribosomal protein S19 | 2312.84 | 1126.30 | -1.22 | <0.001 |
| UYE_RS0128495 | 30S ribosomal protein S3 | 1504.98 | 729.55 | -1.22 | <0.001 |
| UYE_RS0128500 | 50S ribosomal protein L16 | 1382.79 | 645.69 | -1.26 | <0.001 |
| UYE_RS0128505 | 50S ribosomal protein L29 | 1516.26 | 653.40 | -1.30 | <0.001 |
| UYE_RS0128515 | 50S ribosomal protein L14 | 1769.65 | 792.20 | -1.32 | <0.001 |
| UYE_RS0128520 | 50S ribosomal protein L24 | 1951.54 | 882.49 | -1.31 | <0.001 |
| UYE_RS0128525 | 50S ribosomal protein L5 | 1731.63 | 763.90 | -1.33 | <0.001 |
| UYE_RS0128530 | 30S ribosomal protein S8 | 1430.14 | 616.67 | -1.30 | <0.001 |
| UYE_RS0128535 | 50S ribosomal protein L6 | 1723.37 | 799.79 | -1.27 | <0.001 |
| UYE_RS0128540 | 50S ribosomal protein L18 | 1767.93 | 853.58 | -1.23 | <0.001 |
| UYE_RS0128545 | 30S ribosomal protein S5 | 1649.20 | 791.97 | -1.24 | <0.001 |
| UYE_RS0128550 | 50S ribosomal protein L15 | 1863.31 | 1053.12 | -1.02 | <0.001 |

| **Protein folding (Syn6803 Mo_FRL vs. Co_FRL)** | | | | | |
| --- | --- | --- | --- | --- | --- |
| **Gene**  **Symbol** | **Product** | **Mo**  **FRL** | **Co**  **FRL** | **log2Fold Change** | **adjusted p-value** |
| SGL_RS04040 | Hsp20/alpha-crystallin family protein | 433.98 | 2672.60 | 2.02 | <0.001 |
| SGL_RS11615 | ATP-dependent chaperone ClpB | 83.68 | 252.61 | 1.32 | <0.001 |
| SGL_RS13680 | chaperonin GroEL | 38.53 | 104.49 | 1.23 | <0.001 |
| SGL_RS06165 | co-chaperone GroES | 71.40 | 197.67 | 1.18 | <0.001 |
| SGL_RS18085 | molecular chaperone HtpG | 27.42 | 169.61 | 1.17 | <0.001 |
| SGL_RS12750 | molecular chaperone DnaK | 714.51 | 1766.28 | 1.10 | <0.001 |

**Table S9.** Average transcript levels of the genes related to protein folding from the transcriptomic data of Syn6803 monoculture and coculture under FRL. The transcript levels of each gene are normalized as TPM. The average transcript levels were calculated based on three replicates. Only the DEGs are shown in the Table. The locus tag of each gene was assigned based on the annotations in CyanoOmicsDB (http://www.cyanoomics.cn)
